# Extracellular Tau Oligomers Produce An Immediate Impairment of LTP and Memory

**DOI:** 10.1038/srep19393

**Published:** 2016-01-20

**Authors:** M. Fá, D. Puzzo, R. Piacentini, A. Staniszewski, H. Zhang, M. A. Baltrons, D. D. Li Puma, I. Chatterjee, J. Li, F. Saeed, H. L. Berman, C. Ripoli, W. Gulisano, J. Gonzalez, H. Tian, J. A. Costa, P. Lopez, E. Davidowitz, W. H. Yu, V. Haroutunian, L. M. Brown, A. Palmeri, E. M. Sigurdsson, K. E. Duff, A. F. Teich, L. S. Honig, M. Sierks, J. G. Moe, L. D’Adamio, C. Grassi, N. M. Kanaan, P. E. Fraser, O. Arancio

**Affiliations:** 1 Department of Pathology and Cell Biology and Taub Institute for Research on Alzheimer’s Disease and the Aging Brain, Columbia University, 630 W 168th St. New York, NY 10032 USA; 2 Department of Biomedical and Biotechnological Sciences, Section of Physiology, University of Catania, Catania, 95125 Italy; 3 Institute of Human Physiology, Università Cattolica del Sacro Cuore, Rome, 00168 Italy; 4 Department of Biochemistry and Molecular Biology, Institute of Biotechnology and Biomedicine, Universitat Autònoma de Barcelona, Bellaterra, 08193, Spain; 5 Oligomerix, Inc., Oligomerix, Inc., 7 Legion Drive, Suite 101, Valhalla, NY 10595, USA; 6 Department of Neurology, Third Military Medical University, Chongqing, 400042, China; 7 Translational Technology Core Laboratory, Rockefeller University, New York, NY 10065, USA; 8 Department of Chemical Engineering, ASU, Tempe, AZ 85281, USA; 9 Department of Psychiatry, The Mount Sinai School of Medicine, JJ-Peters VA Medical Center, Bronx, NY 10468, USA; 10 Department of Biological Sciences, Columbia University, New York, NY 10027, USA; 11 Department of Neuroscience and Physiology, NYU Langone Medical Center, New York, NY 10016, USA; 12 Department of Microbiology and Immunology, Einstein College of Medicine, Bronx, NY 10461, USA; 13 San Raffaele Pisana Scientific Institute for Research, Hospitalization and Health Care, Rome, 00163, Italy; 14 Department of Translational Science and Molecular Medicine, College of Human Medicine, MSU, Grand Rapids, MI, 49503, USA; 15 Tanz Centre for Research in Neurodegenerative Diseases and Department of Medical Biophysics, University of Toronto, 60 Leonard Avenue, Toronto, Ontario M5T 2S8 Toronto, Canada

## Abstract

Non-fibrillar soluble oligomeric forms of amyloid-β peptide (oAβ) and tau proteins are likely to play a major role in Alzheimer’s disease (AD). The prevailing hypothesis on the disease etiopathogenesis is that oAβ initiates tau pathology that slowly spreads throughout the medial temporal cortex and neocortices independently of Aβ, eventually leading to memory loss. Here we show that a brief exposure to extracellular recombinant human tau oligomers (oTau), but not monomers, produces an impairment of long-term potentiation (LTP) and memory, independent of the presence of high oAβ levels. The impairment is immediate as it raises as soon as 20 min after exposure to the oligomers. These effects are reproduced either by oTau extracted from AD human specimens, or naturally produced in mice overexpressing human tau. Finally, we found that oTau could also act in combination with oAβ to produce these effects, as sub-toxic doses of the two peptides combined lead to LTP and memory impairment. These findings provide a novel view of the effects of tau and Aβ on memory loss, offering new therapeutic opportunities in the therapy of AD and other neurodegenerative diseases associated with Aβ and tau pathology.

Amyloid-β (Aβ) was the focus of most of the studies on Alzheimer’s disease (AD) in the last 20 years. However, Aβ is not the only pathological agent involved in AD. Microtubule Associated Protein Tau (MAPT) is also likely to play a major role in the disease. While Aβ species derive from APP processing, six tau isoforms are derived from alternative splicing of the *MAPT* gene transcript in the adult brain (Fig. S1A). Aβ forms extracellular amyloid plaques, whereas tau forms intracellular insoluble filaments and neurofibrillary tangles (NFTs). In addition, both Aβ and tau form intracellular and extracellular oligomeric species that are soluble pre-fibrillar aggregates^1^,^2^,^3^,^4^, suggesting that the two proteins might share common mechanisms in AD etiopathogenesis.

The prevailing hypothesis in the AD field is that deleterious effects on synaptic function underlying memory loss caused by tau are initiated by Aβ [for a review see^5^]. As AD progresses, tau pathology spreads from the entorhinal cortex in a contiguous, highly selective and highly reproducible fashion, suggesting that extracellular soluble forms of tau transmit pathology from neuron to neighboring neuron [for a review see^6^]. Moreover, once Aβ triggers tau pathology, the disease would progress independent of Aβ^5^. Therefore, therapies targeting Aβ may not be effective once tau pathology is triggered. Nevertheless, tau toxicity does not involve Aβ pathology in tauopathies, suggesting that Aβ is not necessary for tau pathology to occur, and pointing at the need to better clarify the relationship between tau and Aβ.

Here, we investigated whether and how extracellular oligomeric forms of tau (oTau) affect memory and its cellular correlate, long-term potentiation (LTP), either by themselves or in combination with Aβ. Studies with recombinant forms of human tau showed oligomer specific inhibition of LTP and memory formation and were validated using tau derived from AD brains and mice expressing non-mutated forms of human tau (hTau). Collectively, the data demonstrate that a brief exposure to oTau and oAβ either alone or in combination produce an immediate impairment of LTP and memory.

## Results

### Tau is present in the extracellular space where it is released upon neuronal activity

**Figure 1:**
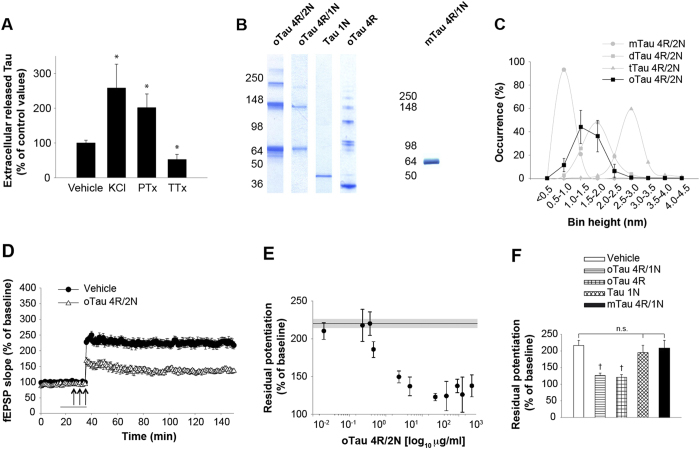
Extracellularly Applied oTau Impairs Hippocampal LTP. (**A**) Soluble tau is released onto the extracellular space upon activity. Neuronal activation by 50 mM KCl or 100 μM PTx increased extracellular tau in primary hippocampal cultures (Mann Whitney: *p < 0.05, n = 7 and n = 4, respectively), while neuronal inactivation by 1 μM TTx reduced it (Mann-Whitney *p < 0.05, n = 10) compared to vehicle (n = 19). All data shown are mean ± SEM. (**B**) Coomassie blue non-reducing SDS-PAGE gel of recombinant oTau 4R/2N, oTau 4R/1N, N-terminal tau (Tau 1N), C-terminal tau (oTau 4R), and monomeric Tau 4R/1N (mTau 4R/1N). (**C**) AFM histogram of recombinant oTau 4R/2N (n = 4). Purified monomeric (mTau), dimeric (dTau), and trimeric (tTau) tau 4R/2N are reported in the figure for comparison. (**D**) Perfusion with 2.29 μg/ml oTau 4R/2N reduced CA3-CA1 LTP (n = 16 slices, ANOVA p < 0.0001 vs. 18 vehicle treated slices). (**E**) Concentration-response curve for the effect of oTau 4R/2N on LTP (n = 6 to 10 slices per concentration). The shaded area corresponds to the average potentiation (continuous line) and the standard error range in vehicle-treated slices in this and the following figures. The residual potentiation was calculated by averaging the last 5 min of LTP at 120 min after the tetanus in this and the following graphs. (**F**) LTP was impaired by 4.29 μg/ml oTau 4R/1N, or equimolar concentrations of C-terminal tau 4R, but not N-terminal tau or mTau 4R/1N (n = 6 to 10 slices per construct; ANOVA p < 0.005, ^†^p < 0.005 vs. vehicle). All data shown are mean ± SEM. See also Supplementary Figure S1.

Memory loss in AD is likely caused by the impairment of processes responsible for synaptic plasticity that are dependent upon neural activity. Given that tau is a major pathological component of AD, we studied the relationship between neuronal activity and tau. We assessed the release of tau into the extracellular medium of 12 day *in vitro* cultured mouse hippocampal neurons exposed for 15 min to vehicle or pharmacological agents that either increase (potassium chloride, KCl, or picrotoxin, PTx) or decrease (tetrodotoxin, TTx) neuronal activity. The treatment with KCl, which stimulates vesicular release, and PTx, which enhances neuronal activity by inhibiting GABA receptors, increased extracellular tau levels (Fig. 1A). By contrast, TTx, which reduces neuronal activity by blocking sodium channels, reduced extracellular tau level (Fig. 1A), suggesting a basal tonic release of tau in the extracellular space. These results confirm that the level of tau released from neurons increases with neuronal activity^7^,^8^.

### Soluble oligomeric forms of recombinant tau impair long-term potentiation (LTP)

The demonstration of activity-dependent tau secretion provided a rationale for investigating the effects of extracellular tau onto hippocampal LTP, an activity-dependent type of synaptic plasticity thought to underlie memory. Following production of purified recombinant tau 4R/2N (the longest adult human CNS isoform of tau, 441 amino acids), the preparation was oligomerized and assessed for oligomerization through non-reducing SDS-PAGE gel, and atomic force microscopy (AFM) (Fig. 1B,C). Additionally, we obtained preparations of oligomeric 4R/1N, oligomer-prone 4R C-terminal, 1N N-terminal tau which did not oligomerize, and monomeric 4R/1N (Fig. 1B). We administered these preparations to mouse hippocampal slices and tested their effect on CA3-CA1 hippocampal LTP. A brief perfusion with oTau 4R/2N for 20 min prior to the theta-burst markedly reduced LTP (Fig. 1D) with an IC_50_ of 0.19 μg/ml (Fig. 1E) without affecting basal synaptic transmission (BST). We obtained similar results with the 4R/1N isoform, as well as the oligomerized C-terminal construct 4R (Fig. 1F). By contrast, monomeric N-terminal construct and monomeric tau 4R/1N did not reduce LTP (Fig. 1F). Together these experiments not only demonstrate that extracellular tau *per se* (without oAβ intervention) can impair synaptic plasticity, but also highlight a direct role for tau oligomerization in LTP impairment. Most importantly, the occurrence of LTP impairment after a brief tau exposure demonstrates that extracellular oTau has a rapid effect on neural activity that may impair plasticity.

### oTau impairs memory formation

**Figure 2:**
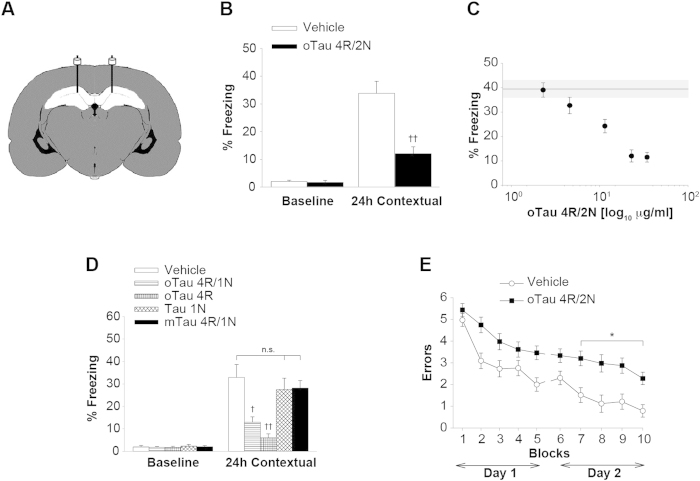
oTau Impairs Memory Formation (**A**) Schematic drawing of cannulas implanted into mouse dorsal hippocampi. Preparations were diluted in a final volume of 1 μl and administered over 1 min bilaterally, 180 and 20 min prior to the training. (**B**) oTau 4R/2N (22.95 μg/ml) impaired contextual memory. Freezing during the training phase (baseline) was not affected by the treatment. Vehicle: n = 18, oTau: n = 11, (24 hrs: ^††^p < 0.001). (**C**) Concentration-response curve for the effect of oTau 4R/2N on contextual memory (n = 10 to 18 mice per concentration). The shaded area corresponds to the average freezing (continuous line) and the standard error range in vehicle-infused mice. **(D)** 21.30 μg/ml oTau 4R/1N, or equimolar concentration of C-terminal tau 4R, but not N-terminal tau 1N or mTau 4R/1N, impaired contextual memory. Vehicle: n = 12, oTau 4R/1N: n = 10, Tau 4R: n = 11, Tau 1N: n = 10, mTau 4R/1N: n = 11, (24 hrs: ANOVA p < 0.0001, ^††^p < 0.001 for Tau 4R vs. vehicle, ^†^p < 0.005 for oTau 4R/1N vs. vehicle). (**E**) oTau 4R/2N (22.95 μg/ml) impaired RAWM performance. 4R/2N: n = 13, vehicle: n = 11. ANOVA p < 0.0001; *p < 0.05 between groups. All data shown are mean ± SEM. See also Supplementary Figure S2.

To extrapolate these electrophysiological findings to memory, we evaluated the effect of extracellular tau onto associative fear memory and spatial memory, two types of memory that are affected in AD patients. In these experiments, oTau 4R/2N (two injections at 180 and 20 min prior to the training) was infused via bilateral cannulas into the dorsal mouse hippocampi (Fig. 2A). This resulted in reduced freezing 24 hrs after the electric shock (IC_50_ = 11.06 μg/ml; Fig. 2B,C). The defect was due to hippocampal impairment as cued fear learning, a type of learning that depends upon amygdala function^9^, was not affected (Fig. S2A). Moreover, the defect was not due to deficits in sensation, as sensory threshold assessment did not reveal any difference between vehicle-infused mice and mice infused with 4R/2N preparation (Fig. S2B). Finally, the defect was due to the presence of oligomers as tau 4R/1N and C-terminal tau 4R constructs which can oligomerize (Fig. 1B) reduced freezing 24 hrs after training, while monomeric N-terminal tau 1N or monomeric tau 4R/1N treatment did not affect freezing (Fig. 2D). This finding is consistent with the observation that accumulation of non-fibrillar soluble tau oligomeric species is associated with memory impairment^10^,^11^.

Short-term spatial memory was tested with the 2-day radial arm water maze (RAWM). Mice infused with oTau 4R/2N (22.95 μg/ml) made a higher number of errors than vehicle-infused mice during the second day of RAWM testing (Fig. 2E). Control trials with a visible platform did not show any difference in speed or latency to reach the platform between the two groups, indicating that oTau infusion did not affect the motility, vision and motivation of mice during RAWM testing (Fig. S2C,D). Moreover, open field testing did not reveal any difference between the two groups indicating that the oTau infusion did not alter mouse exploratory behavior, which might affect animal performance in the memory task (Fig. S2E,F). Collectively, these experiments indicate that a brief exposure to extracellular oTau produces immediate memory impairment.

### The impairment of LTP and memory by recombinant oTau is reproduced by administration of a preparation enriched in soluble tau derived from human AD brain

**Figure 3:**
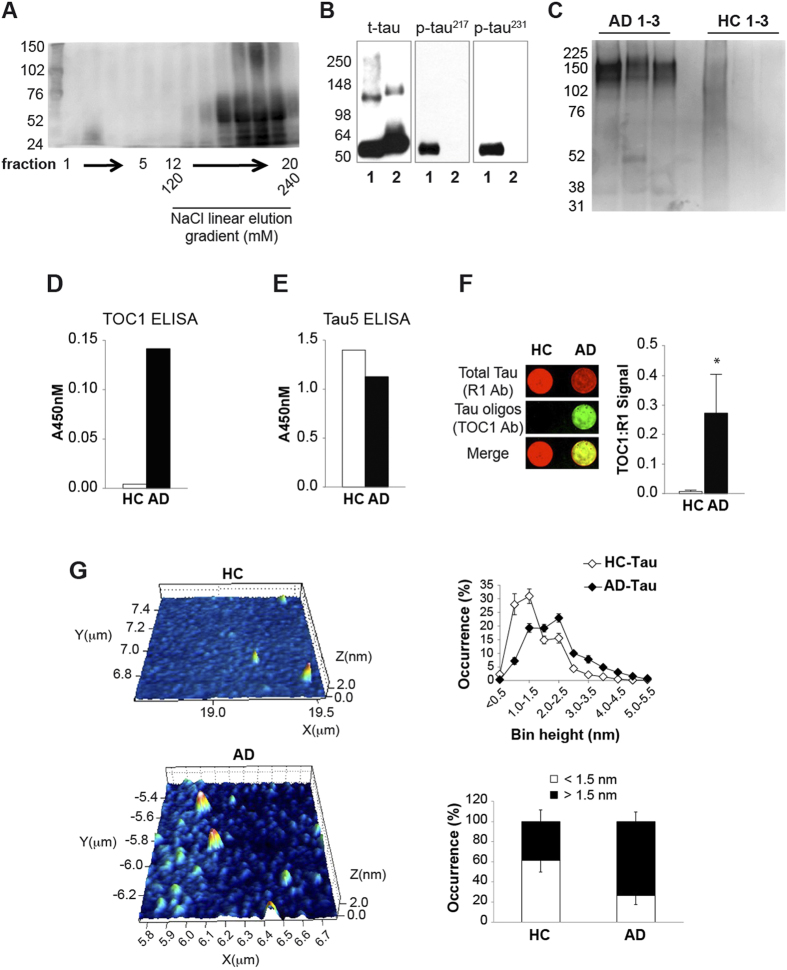
Characterization of Soluble Tau in Human AD Specimens. (**A**) A representative example of non-reducing SDS-PAGE analysis of human tau from AD brain tissue homogenized in a non-reducing buffer. Numbers at the bottom correspond to fraction samples obtained during chromatography. Specimen 15 (Table S1). (**B**) WB of AD-Tau (Lanes 1) vs. total tau (t-tau) by phospho-independent monoclonal Ab HT7, as well as tau phospho-epitope-specific Ab against threonine at amino acid positions 217 (p-tau^217^) and 231 (p-tau^231^). Lanes 2 display analysis from a control recombinant oTau 4R/2N, used as negative control for phospho-epitope specific tau Ab. Specimen 2 (Table S1). (**C**) Non-reducing WB probed with T22 Ab shows bands between 102 and 225 kDa in AD (but not HC) specimens confirming the presence of oligomers in our preparation (AD: 7, 9, 21; HC: 12, 16, 17; Table S1). (**D–E**) Level of tau oligomers in representative HC and AD samples using sandwich ELISAs. Oligomer specific TOC1 Ab shows strong signal in AD, but not in an HC samples (**D**). Tau5 Ab measuring level of total tau shows substantial amounts of total tau protein both in HC and AD samples (**E**) (AD: 9; HC: 17; Table S1). (**F**) Representative TOC1 (green) & R1 (red, total tau) dual color dot blot of HC and AD samples. Results of TOC1 signal normalized to R1 signal, quantified in the graph, indicate significantly more oTau in AD samples compared to HCs (p < 0.05; AD; 7, 9, 21, 22; HC: 12, 13, 16, 17, 23; Table S1). (**G**) AFM suggests the presence of more oligomers in the AD-Tau preparation than in the HC one (bin height 0,5–1.0: p = 0.04; 1.0–1.5: p = 0.06; 2,0–2.5: p = 0.08; 2,5–3.0: p = 0.008). Representative tridimensional images for the two preparations are shown in the color panels. Occurrence of particles is displayed both by 0.5 nm bins (upper graph) and by separating <1.5 nm and >1.5 nm particles (lower graph). Note the absence of particles >5 nm in both preparations (AD: 6, 7, 9, 11, 18, 21, 22; HC: 8, 12-14, 16, 17, 19, 23; Table S1).

As a first approach to establish whether the deleterious effects of recombinant oTau on LTP and memory occur with authentic AD tau species, we obtained preparations enriched in soluble tau from human AD brain specimens (AD-Tau) and control specimens from age-matched individuals (HC). The preparations were characterized prior to performing experiments. First, we confirmed the presence of tau using non-reducing SDS-PAGE and WB with a total tau antibody (Ab) (Fig. 3A,B, Subjects in Table S1). Second, we demonstrated that tau phosphorylation was preserved as shown by immunoblot at T217 and T231 (Fig. 3B), and Mascot database search of LC/MS/MS tandem mass spectrometry data indicating phosphorylation at T181 and S404 both in AD- and HC-Tau (Fig. S3, Table S2). Interestingly, proteomic analysis did not show the presence of amyloidogenic proteins, such as Aβ, synucleins, amylin. Third, we demonstrated the presence of oligomers through T22, a tau oligomer specific polyclonal Ab^12^ (Fig. 3C), and the mouse monoclonal Ab specific for oTau TOC1^13^ as the “capture” Ab in a sandwich ELISA (detection with R1, a rabbit polyclonal pan-tau Ab). TOC1 exhibited reactivity to AD- but not to HC-Tau (Fig. 3D, Table S1). The specificity of this result for tau oligomers was demonstrated by the control ELISA using capture Ab Tau5, a pan-tau antibody for total tau (detection with R1) that showed similar levels of tau in both sets of samples (Fig. 3E, Table S1). Similarly, strong reactivity to AD- but not to HC-Tau, was found with dot blots using TOC1 (Fig. 3F, Table S1) and T22 Ab (data not shown). Finally, when we examined the size of the structures present in our preparations through AFM, we found particles mostly with a 1.5–5 nm diameter, likely corresponding to oligomers, with very few particles <1.5 nm, likely corresponding to monomers, in AD-Tau, (Fig. 3G, Table S1), whereas HC-tau showed a high percentage of ~1 nm diameter structures, and a lower number of particles >1.5 nm (Fig. 3G, Table S1). Interestingly, AFM of both AD- and HC-Tau did not reveal particles >5 nm that might have suggested the presence of large tau aggregates. Collectively, these data confirm the presence of oligomeric tau in our AD-brain derived preparation.

**Figure 4:**
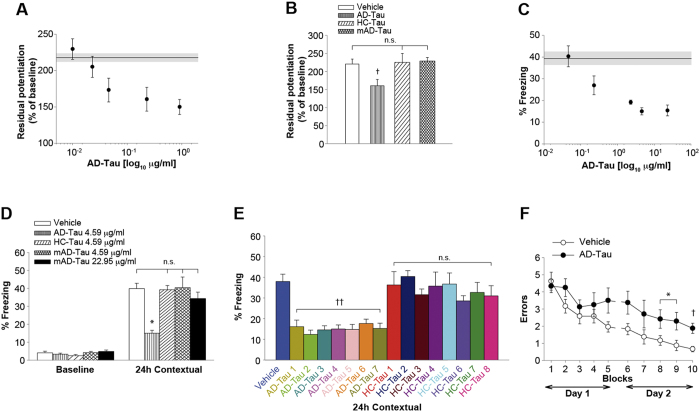
LTP and Memory Impairment by Recombinant oTau is Reproduced by a Preparation Enriched in Soluble Human Tau from AD Patients. (**A**) Concentration-response curve for the effect of AD-Tau on LTP (n = 7-34 slices per concentration). Specimen 15 (Table S1). (**B**) AD-Tau (0.23 μg/ml; n = 13), but not HC-Tau or monomeric AD-Tau (mAD-Tau, n = 8 for both) reduced LTP (vehicle n = 38; ANOVA p < 0.0001 ^†^p < 0.005 vs. all other groups; AD: 15; HC: 13; Table S1). (**C**) Concentration-response curve for the effect of AD-Tau on contextual memory (n = 9 to 13 mice per concentration; specimens 2, 6, 11, Table S1). (**D**) AD-Tau (4.59 μg/ml, n = 9), but not mAD-Tau (both at 4.59 and 22.95 μg/ml; n = 11 and 9, respectively) or HC-Tau (4.59 μg/ml; n = 12), impaired contextual memory. Vehicle (n = 13) ANOVA p < 0.0001, *p < 0.05 vs. other groups (AD: 2, 6, 11; HC: 4, 17, 20; Table S1). (**E**) AD-Tau (4.59 μg/ml) impaired contextual memory [n = 9 for all the specimens except for AD7 (13), AD6 and HC7 (10) and vehicle (20); ANOVA p< 0.0001 ^††^p < 0.001 in AD-Tau groups compared to all other groups. AD: 2, 6, 10, 11, 15, 18, 24, 25; HC: 1, 3–5, 8, 14, 20, 25; Table S1). |(**F**) AD-Tau (4.59 μg/ml) impaired RAWM performance. n = 8 per group, ANOVA p < 0.05, ^†^p < 0.005, *p < 0.05 (Specimens 9, 18; Table S1). See also Supplementary Figure S4.

To test the effects of AD-Tau on LTP we performed a concentration/response curve following 20 min perfusion with AD-Tau. Consistent with the observation that tau interstitial fluid (ISF) levels in healthy individuals have been shown to be 7–15 × 10^−3^ μg/ml^14^, we found an IC_50_ = 0.02 μg/ml (Fig. 4A). By contrast, 0.23 μg/ml HC-Tau did not affect LTP (Fig. 4B). Moreover, if the AD-Tau preparation was incubated with dithiothreitol (DTT), to induce monomerization (mAD-Tau, 0.23 μg/ml), LTP was no longer affected (Fig. 4B). Taken together, these experiments suggest that extracellular oligomeric soluble tau from AD patients impairs LTP.

Similarly to oTau, AD-Tau reduced contextual memory (IC_50_ = 0.20 μg/ml; Fig. 4C), whereas HC-Tau (4.59 μg/ml) or mAD tau (both at 4.59 μg/ml and 22.95 μg/ml) did not (Fig. 4D). Neither amygdala-dependent cued learning nor sensory threshold were affected by AD-Tau, HC-Tau or mAD-Tau (Fig. S4A,B). Most importantly, the marked reduction of contextual learning was confirmed with preparations from 7 AD patients and 8 HC individuals, indicating a high degree of reproducibility across individuals (4.59 μg/ml; Fig. 4E). The similarity between recombinant tau and AD-Tau also held true in short-term spatial memory tasks. Mice that were infused with AD-Tau (4.59 μg/ml) made more errors than vehicle-infused mice during the second day of testing with the RAWM (Fig. 4F). The effect was caused by cognitive impairment because the same animals did not show behavioral differences in control experiments with the visible platform and open field tests that might have interfered with their cognitive assessment (Fig. S4C,F). The results using human tau are consistent with behavioral findings with recombinant tau suggesting that the oligomeric structure is important for memory impairment.

### The impairment of LTP and memory by recombinant oTau is reproduced with 10–11 month old hTau mice, which form oligomers in the absence of NFTs

**Figure 5:**
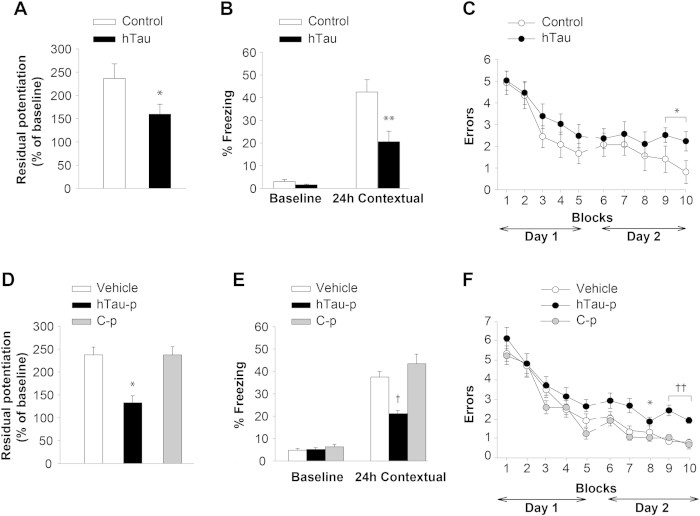
LTP and Memory Impairment by Recombinant oTau is Reproduced by Naturally Produced Human Tau from hTau Mice. (**A**) hTau mice have reduced LTP (hTau: 7 slices/6 mice; Controls: 9/7; ANOVA * p < 0.05). (**B–C**) hTau mice have reduced contextual memory (**B**) and RAWM performance (**C**) (hTau: n = 9, Control: n = 13, fear memory: **p < 0.01, RAWM: ANOVA p = 0.09 *p < 0.05). (**D**) Administration of hTau-p (0.46 μg/ml) reduced LTP, whereas C-p from control mice lacking tau did not (hTau-p: n = 10, C-p: n = 11; vehicle: n = 10 ANOVA *p < 0.05). (**E–F**) hTau-p (4.59 μg/ml) reduced contextual memory (**E**) and RAWM performance (**F**) whereas C-p did not (hTau-p: n = 10; C-p: n = 11, vehicle = 10 fear memory: ANOVA p <0.001, ^†^p < 0.005 vs. C-p, RAWM: ANOVA p < 0.0001 *p < 0.05 and ^††^p < 0.001 vs. C-p). All data shown are mean ± SEM. See also Supplementary Figure S5.

As an additional control method alternative to the use of soluble tau derived from human AD brain to further validate findings with recombinant oTau, we repeated the electrophysiological and behavioral studies in hTau mice naturally forming oTau from 6 months of age prior to NFT appearance and their control littermates lacking tau^15^. Neurons of 10–11 month old hTau mice showed hyper-phosphorylated tau by immunohistochemistry, but no NFTs were seen on Bielschowsky staining (Fig. S5A). However, aged (18 month) hTau mice had hyper-phosphorylated tau and NFTs (Fig. S5A), consistent with the interpretation that 10–11 month-old hTau mice were in a pre-tangle state. Interestingly, LTP and both contextual and spatial memory were impaired compared to control animals at 10–11 months (Fig. 5A–C). Furthermore, the two groups of animals did not show any difference in BST (Fig. S5B), cued conditioning, sensory threshold, visible platform and open field performance (Fig. S5C–H). Most importantly, a preparation enriched in soluble human tau obtained from the hTau mouse cortex (hTau-p), but not from tau-lacking control mice (C-p) (Fig. S5I), reduced LTP, contextual and spatial memory (Fig. 5D–F) without affecting cued conditioning, sensory threshold, visible platform and open field performance. Collectively, these experiments with endogenously produced oTau from a transgenic mouse model of AD confirm impairment of LTP and memory by exogenous recombinant human tau and human tau derived from AD brains.

### oTau acts concurrently with oAβ to impair LTP and memory

**Figure 6:**
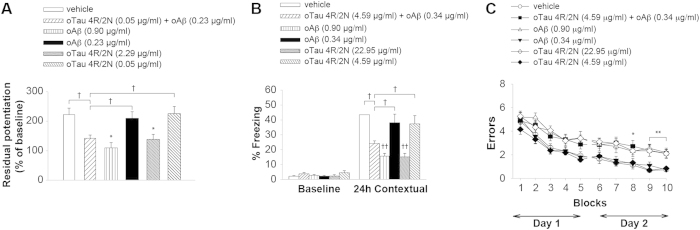
oTau can Act Concurrently with oAβ to Impair LTP and Memory. (**A**) Subthreshold doses of oTau 4R/2N (0.05 μg/ml) plus oAβ (0.23 μg/ml) reduced LTP (n = 11), whereas the same concentrations of the two oligomers alone did not. Vehicle n = 9, oTau (0.05 μg/ml), oAβ (0.23 μg/ml), and oTau (2.29 μg/ml) alone n = 8, oAβ (0.90 μg/ml) n = 7. ANOVA p < 0.0001; ^†^p < 0.005, *p < 0.05 vs. vehicle. (**B**) Subthreshold doses of oTau 4R/2N (4.59 μg/ml) plus oAβ (0.34 μg/ml) impaired contextual memory (n = 13), whereas the same concentrations of the two oligomers alone did not. Vehicle n = 10, oTau (22.95 μg/ml) n = 13, oTau (4.59 μg/ml) n = 10, oAβ (0.90 μg/ml) n = 11, oAβ (0.34 μg/ml) n = 11 (24 hrs: ANOVA p < 0.0001; ^†^p < 0.005, ^††^p < 0.001 vs. vehicle). (**C**) Subthreshold doses of oTau 4R/2N (4.59 μg/ml) plus oAβ (0.34 μg/ml) (n = 11), but not the same concentrations of the two oligomers alone, impaired RAWM performance (oTau n = 11, oAβ n = 10). Vehicle n = 11, oTau (22.95 μg/ml) n = 10, oAβ (0.90 μg/ml) n = 9. ANOVA p < 0.0001 *p <0.05 **p < 0.01 vs. vehicle, or 4.59 μg/ml oTau 4R/2N, or 0.34 μg/ml oAβ). All data shown are mean ± SEM. See also Supplementary Figure S6.

Both tau and Aβ are β-sheet forming proteins with propensity for oligomerization and close association with membranes suggesting a common toxicity mechanism^16^. Thus, we decided to determine if the combination of tau and Aβ oligomers - at subthreshold doses that give no significant impairment for LTP and memory for each alone according to their respective dose/response curves - impairs LTP and memory. Slices perfused for 20 min with subthreshold doses of oTau 4R/2N (0.05 μg/ml) plus oAβ_42_ (0.23 μg/ml) prior to inducing LTP revealed a marked reduction of potentiation compared to vehicle treated slices or slices treated with the same low doses of tau or Aβ alone (Fig. 6A). In interleaved experiments high amounts of oTau 4R/2N (2.29 μg/ml) or oAβ (0.90 μg/ml) alone were capable of affecting LTP (Fig. 6A). Similarly, the combination of 4.59 μg/ml oTau 4R/2N plus 0.34 μg/ml oAβ prior to electric shock for fear conditioning or training for RAWM affected the two forms of memory (Fig. 6B,C). The same low doses of oTau and oAβ alone did not affect memory, whereas high concentrations of oTau 4R/2N (22.95 μg/ml) or oAβ (0.90 μg/ml) alone reduced memory. Moreover, we did not observe any behavioral differences between groups of mice with cued conditioning, sensory threshold, visible platform and open field (Fig. S6A–F). Collectively, these experiments demonstrate that tau and Aβ oligomers can act concurrently.

### oTau enters neurons

**Figure 7:**
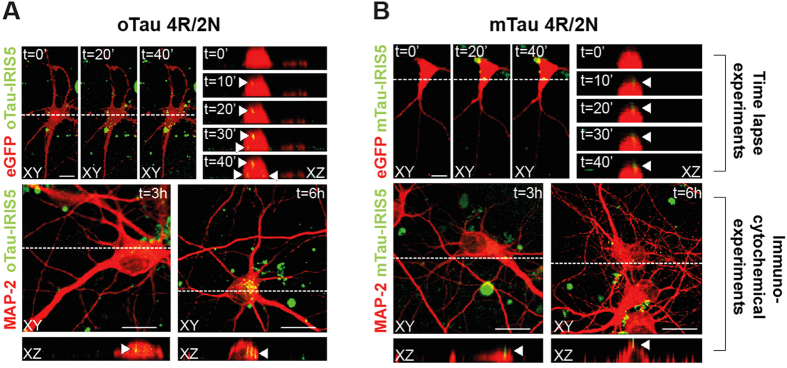
oTau, but not mTau, is internalized in neurons. (**A,B**) Representative examples of mouse hippocampal neurons exposed to 5 μg/ml oTau 4R/2N (**A**) or mTau 4R/2N (**B**). oTau or mTau were labeled at the N-terminus with the fluorescent dye IRIS-5-NHS. Upper panels show data obtained in time-lapse confocal experiments performed on eGFP-expressing neurons at DIV14. Data shown in the lower panels were obtained from immunocytochemical experiments performed after 3–6 hr tau application in neurons immunolabeled for the microtubule associated protein-2 (MAP-2). Colors of eGFP, MAP-2 and IRIS-5 were inverted to optimize image visualization. (**A**) oTau was clearly internalized (in yellow) within 30-min of application as shown in the XZ cross-sections from the Z-stack acquisitions (panels on the right). After 3 and 6 hrs, oTau internalization, but not mTau (**B**) was much more intense, as revealed by immunocytochemistry (lower panels, and XZ cross sections on the bottom). Time lapse confocal experiments: n = 10 both for oTau and mTau; Immunocytochemistry: n = 40 per each time point (3 and 6 hrs) and condition (oTau and mTau). Scale bars: 25 μM.

All our experiments have been performed through exogenous application of tau, yet molecular memory mechanisms affected by the protein are intracellular. Our next goal was therefore to establish a possible avenue between extracellular oTau and intracellular events. Misfolded tau has been shown to be internalized by cells *in vitro*^17^. For instance, uptake of low molecular weight tau species has been found in cultured neurons through endocytosis^18^. In light of the profound effect that tau exposure has on LTP and memory, we decided to perform optical measurements in mouse hippocampal cultures treated with 5 μg/ml oTau 4R/2N in order to determine whether also our tau 4R/2N preparation can be internalized in neurons. A time-lapse confocal imaging study demonstrated that oTau 4R/2N conjugated to IRIS-5 penetrates the cell membrane of cultured eGFP-expressing neurons after 20 min of exposure (yellow staining) (Fig. 7A, upper panels). By contrast, mTau 4R/2N conjugated to IRIS-5 could not be visualized up to the last time point investigated after 40 min of exposure (Fig. 7B, upper panels). We reached similar conclusions when we used immunocytochemistry on MAP-2 labeled neurons that were exposed to oTau 4R/2N conjugated to IRIS-5 for 3 or 6 hrs prior to fixation. Intracellular tau (yellow) was visible at 3 hrs, and the staining acquired a punctuated pattern at 6 hrs suggesting that tau localizes to vesicular structures^19^ (Fig. 7A, lower panels). By contrast, there was no internalized tau after mTau 4R/2N exposure both at 3 and 6 hrs (Fig. 7B, lower panel). These findings demonstrate that oTau crosses cell membrane. By contrast, consistent with the previous study on primary mouse hippocampal cultures^18^ (but see also another study on SH-SY5Y cells^19^), tau monomers do no penetrate cells within the time frame investigated in the present study. Taken all together these findings support the possibility that oligomeric extracellular tau acts onto molecular mechanisms of learning and memory after penetration inside the cells.

## Discussion

The accumulation of protein aggregates in the brain is a common process in neurodegenerative diseases, each disease having its own specific aggregating proteins and distribution. Recently, the focus has shifted from large protein precipitates to small, very soluble aggregates called oligomers, as they appear more acutely toxic than large insoluble aggregates. Our studies establish a novel model through which oTau causes synaptic dysfunction and memory loss. Extracellular tau oligomers, recombinant or extracted from AD brains or naturally produced from hTau mice, effectively and consistently induce key features of AD including synaptic dysfunction and memory loss, whereas tau monomers produce no deleterious effects. These effects have a very fast onset as they are present within a few minutes from the application of the oligomers. Additionally, they occur with tau alone, or in combination with oAβ as sub-toxic doses of oTau affect LTP and memory when paired with sub-toxic doses of oAβ.

We found that recombinant extracellular oTau *per se* is capable of negatively affecting LTP and memory. This discovery was validated by our examination of soluble AD-Tau as well as experiments with hTau mice expressing non-mutated forms of human tau and forming oligomers prior to NFTs. Importantly, monomerized AD-Tau preparations containing post-translational modifications (PTMs), or purely monomeric tau recombinant forms, either in full length or as 255 amino acid long N-terminal fragment, failed to produce synaptic dysfunction and memory loss. In contrast, the oligomerized C-terminal fragment of recombinant protein impaired LTP and memory. This is consistent with the observation that tau multimer levels correlate with memory loss in the rTg4510 mouse model^10^. The C-terminal portion of tau containing the microtubule binding repeats is prone to aggregate as it has β-sheet forming hexapeptide motifs that self-interact, as well as cysteines that form disulfide linkages to stabilize the interactions. The N-terminal portion of tau used in our experiments does not have regions of tau self-interaction that have been characterized, and does not have a tendency to form aggregates upon incubation. Therefore, it was used as an additional control for non-aggregated tau. However, this does not exclude that other types of N-terminal fragments of a different length than the one used in our studies, either in a monomeric or oligomeric form, might be toxic. Consistent with this possibility, different effects have been reported according to the type of N-terminal fragment utilized. For instance, it has been found in cerebellar granule cells that the short N-fragment 1–44 is particularly toxic, whereas overexpression of longer fragments (1–230) and tau (1–441) blocks apoptosis^20^. Of note, our studies were performed with a 255 amino acid fragment that is consistent with lack of toxicity observed in cerebellar granule cells with tau fragments of similar length^20^. Moreover, an N-terminal motif in tau, called the phosphatase-activating domain (amino acids 2–18), can impair fast anterograde axonal transport when abnormally exposed in pathological forms of tau^21^,^22^,^23^. Together these data suggest that several routes of tau-mediated toxicity may exist and that different parts of the protein are involved. The current studies suggest that the N-terminus (at least amino acids 1–255) is insufficient to disrupt LTP, but this part of the tau protein may be involved in other toxic mechanisms. Nevertheless, the observation that extracellular tau oligomers are likely to play a key role in synaptotoxicity is important for better understanding the role of tau in disease.

An important aspect of our studies is that the oTau effect on LTP and memory is rapid (i.e. as soon as 20 min after exposure). The implication of this observation is that memory loss in AD patients is likely to be due to a modification of the dynamic and fast processes underlying plasticity and memory formation that are being inhibited by oligomers. This is consistent with the widely accepted notion that synaptic strengthening is central to memory formation, and dementia could result from altered strengthening of synapses. As a consequence, our studies suggest that AD therapeutics acting on tau oligomers should produce beneficial effects on memory at any disease stage, as the most likely scenario for their action is that molecular mechanisms underlying plasticity and memory are continuously deranged by the oligomers as the disease evolves.

Both the C-terminal fragment and full-length oligomerized tau impaired LTP and memory. This begs the question of whether these forms of tau are secreted into the extracellular space, an essential requirement for our observations to be biologically relevant. To this end, secretion of different tau fragments has been investigated on synaptosomes prepared from cryopreserved human postmortem tissue^24^. This study showed that an extended tau form (~55 kDa), the truncated tau fragment (~20 kDa), as well as other N-terminal tau fragments (35–50 kDa) are released following depolarization. Only 15–25% of synaptosomes were immunoreactive for the tau 46 C-terminus antibody; however, the tau 46 antibody fails to recognize several secreted C-terminal tau fragments^25^. Of note, this study also showed a significant increase in C-terminal tau immunoreactivity in AD compared to aged normal synapses^24^, suggesting that 4R tau cleavage fragments are efficiently secreted, especially in AD. In summary, both full-length tau and C-terminal tau appear to be secreted outside cells, and therefore, our findings are biologically relevant.

We have found a marked reduction of LTP following slice perfusion with recombinant tau oligomers. This observation is consistent with the finding of an impaired long-term depression in hippocampal slices taken from an inducible mouse model expressing the tau repeat domain with the pro-aggregant mutation ΔK280^26^. Another study, in turn, showed a reduction of short-term potentiation with no significant effect on LTP by recombinant tau^27^. A possible explanation for the difference between our findings and those of this study that was also performed with recombinant tau^27^ is linked to the different method of tau oligomerization. In the previous study^27^, tau was oligomerized through seeding with Aβ, whereas our oligomerization method relied upon reduction and oxidation of cysteine residues. Tau oligomerization is typically achieved through different protocols such as addition of heparin, heparan sulfate, polyunsaturated fatty acids, RNA, or quinones^28^,^29^,^30^,^31^,^32^ to drive it. These different protocols, although very efficient in producing tau oligomerization, are likely to result in a variety of species^1^ which in turn might be responsible for different levels of toxicity or no toxicity at all. Indeed, as reported^33^, the kinetics of aggregation differs among protocols and the obtained tau species are not necessarily equivalent both in terms of size and biophysical properties. A standardized method is necessary for the production of tau. Although our current knowledge does not allow us to define the tau species that is/are toxic, our method offers the advantage of producing oligomers that efficiently and quickly affect both synaptic function and memory, two key features of the disease.

We have discovered that sub-toxic doses of oTau affect LTP and memory when paired with sub-toxic doses of oAβ. It is therefore possible that either the two oligomeric proteins act at the same level in the chain of molecular events leading to AD, or that they act on different targets that later converge on a common molecular downstream indirect target. This observation combined with the finding that the two peptides alone lead to LTP and memory impairment, independent of the presence of high concentrations of the other, suggests that it is not necessary to evoke the presence of elevated levels of Aβ to initiate the molecular mechanisms underlying synaptic dysfunction and memory loss in AD. Consistent with this conclusion, tau toxicity does not involve Aβ pathology in tauopathies. In future experiments we will study whether other β-sheet forming proteins (i.e. synuclein, amylin) act concurrently with tau and/or Aβ to affect LTP and memory. We will also investigate whether the combined action of oTau and oAβ is synergistic or additive, with a mechanism dependent upon interaction between tau and Aβ oligomers prior to their binding with a common receptor, or as a consequence of their binding with the receptor. Nevertheless, the main observation of this manuscript showing that exogenous oTau and oAβ concurrently disrupt synaptic plasticity and memory formation is clear and has important implications for understanding AD pathology.

We have found another interesting parallelism between tau and Aβ. Similar to Aβ^34^,^35^, tau can be secreted from hippocampal neurons in the extracellular space in a fashion that is regulated by neuronal activity via a Na^+^-channel dependent mechanism. This finding is consistent with the observation that tau is secreted into the extracellular space^25^ in an activity-dependent fashion^7^,^8^, and supports the concept that soluble oligomeric tau, whether secreted in oligomeric form *per se* or oligomerized outside the cells, can be detected in CSF. Furthermore, this finding is in agreement with the hypothesis that release of intracellular tau into the extracellular space promotes seeding in other brain cells^17^,^36^,^37^,^38^,^39^,^40^. Interestingly, our data unravel a tonic release of tau in the extracellular space, given that TTx-induced block of voltage-gated Na^+^-channels dramatically decreases tau secretion. This data is different from a study on ISF tau in which TTx failed to affect tau levels differently than Aβ levels^8^. However, this study used *in vivo* microdialysis, a technique that relies on protein turnover rate (half-life of ~11 days for tau vs. ~2 hrs for Aβ)^8^, and therefore precluded the investigators from assessing the role of Na^+^ channels in tau release. One can speculate that the tonic release of tau performs a trophic action at the synapse. Future studies outside the scope of the present work will be necessary to demonstrate this.

In agreement with previous data showing tau internalization^17^,^18^, we have found that tau oligomers enter neurons after 20 min of exposure. Consistent with these findings, tau oligomers produce an immediate impairment of LTP and memory. Thus, a straightforward scenario that will be investigated in future experiments is that once oligomers are internalized they are detrimental to second messenger cascades involved in synaptic strengthening and memory formation.

Another important aspect of our studies is that synaptic and memory dysfunctions did not require the presence of NFTs. Indeed, we have shown that tau oligomer exogenous administration produced its deleterious effects in normal mice, and hTau mice display LTP and memory impairment in the presence of tau oligomers prior to the appearance of NFTs. This is consistent with work demonstrating that tau oligomer-specific antibodies (e.g. TOC1) label tau pathology in a pre-tangle state^41^ prior to coalescing into NFTs^13^,^42^,^43^. Altogether, these findings suggest that tau oligomers precede the formation of NFTs, underlying subtle changes in synaptic function that are responsible for amnestic symptoms. However, this does not mean that insoluble tau precipitates have no pathogenic role in neuronal dysfunctions. Their invariant accumulation may signify that they serve as reservoirs of tau both in fragmented or low-n oligomeric forms, with NFTs serving as a protective mechanism sequestering toxic soluble tau species^44^,^45^. Alternatively, NFTs might play a role in other aspects of the disease (i.e. spread within the brain), independently of a direct effect on memory.

Our findings with recombinant tau were validated through two alternative sets of experiments performed either with a preparation enriched with tau derived from human specimens or with hTau mice. The method we used to extract tau from the specimens assured a high yield for the protein and preserved its phosphorylation status. Moreover, it excluded amyloidogenic proteins, such as Aβ, synucleins, amylin, as confirmed through proteomic analysis of the samples. However, it did not assure absolute purity of the preparations, in the hypothesis that other oligomerized proteins were responsible for the detrimental effects on LTP and memory. To overcome this drawback, we further purified our AD-Tau preparation via immunoaffinity in a limited set of LTP and fear conditioning experiments confirming our results from preparations obtained without the last immunoaffinity step. Most important, given that it is highly unlikely that one can obtain a human preparation absolutely devoid of any other molecule, regardless of the method used, yet preserving the post-translational status of tau and the putative oligomeric toxic form of tau, we used hTau mice. These animals were useful for demonstrating the role of tau in the electrophysiological and behavioral defects because at 10–11 months they form tau oligomers in the absence of NFTs^15^. Thus, they can be used as a source of human tau oligomers, whereas their littermates lacking tau can be utilized as a negative control. Strikingly, either 10–11 month old hTau mice or a preparation enriched in human tau extracted from their brains reproduced the same findings as with AD-tau and recombinant tau. Obviously, one cannot exclude that expression of the tau human transgene generates other oligomeric proteins that might, in turn, be responsible for the effect onto LTP and memory. In this unlikely scenario, oTau would be still relevant to the disease etiopathogenesis.

Our preparations either derived from human specimens or from cortices of hTau mice were obtained by homogenizing cortical tissue. This prevented us from distinguishing between the intracellular and extracellular pool of tau. We chose using cortical tissue because the method assured sufficient amounts of tau for performing our experiments, whereas other sources of the protein such as CSF in which tau clearly belongs to the extracellular pool, would have not permitted to recover enough protein for performing our experiments. Moreover, our method did not allow us to distinguish between different forms of tau, some of which might be toxic and others not. To address these issues, we confirmed findings with exogenous tau using hTau mice that are likely to reproduce more faithfully than brain extracts what happens in the diseased brain, as these animals naturally produce human tau. Moreover, we obtained a similar finding using recombinant tau. In summary, we reached the same conclusions using four different methods, including human recombinant tau, tau derived from human specimens, human tau derived from hTau mice, and *in vivo* hTau mice. Thus, the main observations of this manuscript showing that exogenous tau oligomers impair LTP and memory are clear and have important implications for the treatment of diseases characterized by abnormal tau elevation.

Recombinant tau or tau extracted from a specimen might be different than tau released into the extracellular space *in situ*. To alleviate concerns regarding differences between recombinant tau and extracted tau used in our experiments and secreted tau, it should be noted that release of aggregated forms of tau has already been shown^19^. Moreover, the same study reported results suggesting an uptake of monomeric and/or small oligomeric species formed by full-length tau or the C-terminus. Finally, tau aggregates formed in one cell were found to be released into the extracellular space, gain entry into neighboring or synaptically connected cells, and trigger further aggregate formation via templated conformational change^46^. Thus, it is reasonable to assume that our recombinant tau oligomers, as well as oligomers derived from human and hTau mouse brains, have relevance to human pathology, even if we could not use naturally secreted tau in the current studies.

While the present investigation is not aimed at evaluating the modalities and the extent of tau propagation, our data support and extend the notion that the presence and diffusion of extracellular tau is potentially harmful. The exact mechanism by which extracellular tau may potentially interact with exposed targets on the cell surface or after it enters inside the cell is still matter of intense debate and investigation^47^. Secretion of tau is not fully understood yet and there is no consensus regarding the mechanisms solely involved in physiological release or in pathological conditions. Evidence supports the release of both monomeric and oligomeric forms of tau, as well as tau fragments, with variegated levels of toxicity, depending on concentration, size of the oligomer or tau fragment, conformation, and *prionoid* activity upon internalization. Importantly, our data show a robust acute effect of tau oligomers onto synaptic plasticity and memory suggesting this phenomenon occurs independently of pathology propagation. In this instance, oligomerization seems important for plasticity and memory impairment, but it is not essential *per se* for secretion and propagation, and therefore cannot be generalized to all aspects of AD. Nevertheless, the process of tau spreading throughout the neural circuitry involved in memory would provide a means for extracellular oligomers to impair LTP and memory to a progressively growing extent.

Tau and Aβ oligomers produce common biochemical neuronal modifications relevant for molecular mechanisms of gene transcription involved in memory formation. This is important in terms of translational significance because the most compelling and relevant outcome from our study is that we have highlighted a new model for oligomer-induced toxicity in AD, modifying the classical view that oAβ triggers molecular modifications responsible for amnesic changes in the disease via tau. A unifying hypothesis for the disease origin is that the oligomeric conformation of proteins involved in AD, such as Aβ and tau, is toxic, independent of the type of protein forming the oligomers. Therapies acting onto a common target (possibly in sites of interaction with the oligomers instead of the classical β- and γ-secretase sites), or downstream of it, might therefore represent a valid and effective strategy for developing therapeutics.

## Methods

### Animals

All protocols involving animals were approved by Columbia University, NYU and Università Cattolica del Sacro Cuore, and the respective Institutional Animal care and Use Committee (IACUC); experiments involving animals were performed in accordance with the relevant approved guidelines and regulations. C57BL/6J, hTau (Jackson Lab Stock #005491), eGFP (Jackson Lab Stock #07075) mice and their littermates were obtained from breeding colonies kept in the animal facility of Columbia University, NYU, and Università Cattolica del Sacro Cuore. They were 3–4 months of age except for hTau mice which were 10–11 months old, and newborn mice for cell cultures. The methods for genotyping the colonies have already been described^48^,^49^. All mice were maintained on a 12 hr light/dark cycle (with lights on at 6:00 A.M.) in temperature and humidity-controlled rooms of the animal facilities.

### Measurement of Tau Release in Primary Neuronal Cultures

Cell cultures were prepared from hippocampi of 0- to 1-day old newborn mice (C57B6/JL) as described^50^. At 12 DIV, cell medium was discarded and the dishes were washed three times with warm HBSS medium to remove debris and neurotransmitters or proteins. Then cells were treated with either vehicle, or 50 mM KCl, or 100 μM picrotoxin (PTx), or 1 μM tetrodotoxin (TTx) in Artificial Cerebro-Spinal Fluid (ACSF) (NaCl 124 mM, KCl 4.4 mM, Na_2_HPO_4_ 1 mM, NaHCO_3_ 25 mM, Glucose 10 mM, CaCl_2_ 2 mM, MgCl_2_ 2 mM) for 15 minutes (37 °C) prior to harvesting both extracellular medium and cells. Cell death was assessed through trypan blue staining^51^. Upon collection, ACSF samples were flash frozen and stored at −80 °C until analysis through an innovative ELISA test coupled to a derivative of ruthenium which generates chemiluminescence by electrical pulses produced by the Sector Imager Reader (MSD, MesoScale, MD), as reported elsewhere^52^,^53^. Briefly, 96-well plate in the MesoScale Tau Kit was blocked by adding phosphate buffer containing 5% IgG-free bovine serum albumin and 1% Tween 20 for 1 hr. Anti-Tau antibody (4/53) was diluted in low cross buffer (MSD) containing 0.1% BSA and 0.1% Tween 20, and then incubated 1 hr at room temperature. The plates were then washed three times with PBS buffer containing 1% Tween. For detection of bound antibody, the anti-Tau SULFO-TAG-detection antibody was added after washing, at a final concentration of 10 nM and plates were incubated for 1 hr at room temperature. After addition of MSD reading buffer, tau signals were detected by electrochemoluminescence using the MSD SECTOR-Imager 2400 as described above. The intra-assay coefficient of variation (%) for total tau was 11.9.

### Preparation of Recombinant Tau

The cDNA for tau 4R/1N (412 amino acid isoform) was purchased from OriGene and subcloned into the bacterial expression vector pET21B to produce the tau protein. Similarly, the amino (amino acids 1–255) and carboxyl-terminal tau fragments (amino acids 256–441) were subcloned from this cDNA. The tau 4R/1N construct and the derivative subclones have a C-terminal 6x His-tag. The tau 4R/2N construct was a gift of Dr. Furukawa (University of Yokohama, Japan)^54^. The plasmid was transfected in *Escherichia coli* (Rosetta), and cells were streaked on LB agar ampicillin plates and a single colony was picked and grown overnight in LB broth with glucose and 100 mg/ml carbenicillin. Protein expression was induced with 1 mM IPTG for 8 hrs at which time cells were pelleted at 4 °C by centrifugation at 6000 g. Pellets were stored overnight at −80 °C. After a freeze-thaw cycle, cells were lysed in a 2% Triton X-100 phosphate-buffered saline and with a protease inhibitor mixture (Complete, EDTA-free; Roche Diagnostics. Streptomycin sulfate was added to precipitate DNA. After centrifugation, 100 mM NaCl was added to the supernatant and heated at 100 °C for 15 min. The precipitate was removed by centrifugation. The first step of purification for the C-terminal anionic construct used a nickel column with His-bind resin. The supernatant was loaded on His-Spin Protein miniprep columns (Zymo Res.) and eluted with phosphate buffer containing 300 mM NaCl plus 250 mM imidazole. Eluted tau was then buffer exchanged for the protein preparations into 50 mM Tris-HCl pH 7.4 via Amicon Ultra Centrifugal Devices (Millipore). Protein concentration was determined with BCA assay (Thermo Sci.). Monomer, dimers and trimers were purified from the oligomer mixture by size fractionation and analyzed by non-reducing SDS-PAGE^55^. Size fractionation followed by SDS-PAGE was also used to separate monomers and oligomers from the Tau 4R/1N preparation^55^.

### Tau Oligomerization

Tau was monomerized by treatment with 5 mM dithiothreitol (DTT) and 5 mM EDTA. Oligomerization was achieved via introduction of disulfide bonds through incubation with 1 mM H_2_O_2_ at RT for 20 hrs. Upon oligomerization tau was buffer exchanged to remove excess chemicals. Any insoluble material was removed by ultracentrifugation at 110,000 × *g* at 4 °C for 30 min. Tau protein concentration was determined from the absorption at 280 nm with an extinction coefficient of 7450 cm^−1^ M^−1^. Given that tau preparations contain a mix of monomers and different size oligomers, and tau conformation may change between initial preparation and final experimental conditions, tau concentration was expressed in μg/ml.

### Assessment of Immunoreactivity for Tau and Neuronal Proteins

oTau 4R/2N and human-specimen derived tau were run on precast 3–8% gradient polyacrylamide Tris-acetate gels (Invitrogen). Proteins were transferred to nitrocellulose membrane (Millipore). Tau immunoreactivity was detected using anti-total tau polyclonal antibody (1:2000; Epitomics), and phosphospecific polyclonal antibodies against tau [p-tau^217^] and [p-tau^231^] (Invitrogen). oTau was characterized through the conformational antibodies T22 (Millipore) and TOC-1^13^,^42^,^43^,^56^. Duplicate blots were first performed with the phosphorylated tau antibodies and re-used with for total tau analysis.

### Atomic Force Microscopy (AFM)

AFM sample preparation and analysis was performed as previously described^55^,^57^ (see Supplementary Methods for a detailed description).

### Electrophysiological Studies

Hippocampal slices were cut with a tissue chopper and recorded as described^58^ (see Supplementary Methods for a detailed description).

### Behavioral Studies

Intracerebral tau or Aβ infusion, and behavioral tasks including fear conditioning, RAWM, sensory threshold, visible platform and open field were assessed as described^58^,^59^,^60^ (see Supplementary Methods for a detailed description).

### Extraction of Human Tau

For obtaining a preparation enriched in soluble human tau we used the prefrontal/frontal cortex of AD patients and HCs (Table S2), as well as cortices from hTau mice. Human tissue was provided by the New York Brain Bank–The Taub Institute, Columbia University, and the VAMC NY. The tissue was prepared as previously described^61^ with the modification that tau solubilization was achieved without the use of detergents (allowing the selective recovery of the soluble fraction), and immunoaffinity column for the final step (to achieve high quantities of protein: 0.006–0.1% mass of frozen brain tissue), except for a few control experiments in which we included immunoaffinity, confirming LTP and contextual memory impairment by the preparation. Furthermore, all molecules below 10 kDa were filtered out through Sartorius Vivaspin-Turbo-15 for the final purification step. Advantages of this method are that the acidic homogenization buffer containing 1% perchloric acid allows the removal of DNA and the vast majority of other proteins than tau, whereas it preserves the tau phosphorylation status^62^. Reductant was used during extraction and fractions containing monomeric tau were pooled, concentrated and buffer exchanged into 50 mM Tris-HCl pH 7.4. Then, the preparation was oligomerized according to the method for tau oligomerization described above.

### Proteomic Analyses

Extracted tau protein^61^ was prepared for mass spectrometry by alkylation of cysteines, digestion with trypsin and analyzed with a NanoAcquity UPLC and Synapt G2 quadrupole-time-of-flight HDMS mass spectrometer (Waters) as described previously^63^. Spectra were collected by data-dependent acquisition as previously^63^, except that the survey scan time was 0.25 s, five ions were selected after a single survey scan, and advanced charge state peak detection was used with collision energy ramping (12–40 V start and 20–60 V end). The Mascot database search program (Vers. 2.4) (Matrix Science, London, UK) was used for protein and peptide identifications. Observed masses were searched by Mascot against the NCBI nr protein database of 08/14/13 (31,351,517 sequences; 10,835,265,410 residues) with *Homo sapiens* taxonomic filter (251,429 sequences). Search parameters included fixed modification of carbamidomethyl (C), variable modifications of oxidation (M), phospho (STY), peptide mass tolerance ± 0.01 Da and fragment mass tolerance ± 0.02 Da with decoy search enabled.

### Enzyme-linked immunosorbent assay (ELISA)

Tau5 (total tau, human brain samples), or TOC1 (tau oligomers) were used as the capture antibody and R1 tau antibody (a polyclonal rabbit tau antibody) for detection of bound tau. All steps were performed at room temperature, 200 μl/well was used for rinsing and blocking steps, and 50 μl/well for all other steps. Capture antibodies were diluted (Tau5, 1 μg/ml; TNT1 and TOC1, 2 μg/ml) in borate saline (100 mM boric acid, 25 mM sodium tetraborate decahydrate, 75 mM NaCl, 250 μM thimerosal) and incubated in high binding ELISA microplates (Corning, #3590) for 1 hr. Plates are then rinsed twice with ELISA wash buffer (100 mM boric acid, 25 mM sodium tetraborate decahydrate, 75 mM NaCl, 250 μM thimerosal, 0.4% bovine serum albumin and 0.1% tween-20) and blocked with ELISA wash containing 5% non-fat dried milk for 1 hour. Each well was rinsed 2 times and samples added to the well for 1.5 hrs. *In vitro* tau aggregation samples were diluted in Tris Buffered Saline (TBS) to 25 nM and human brain extracts to a final total protein concentration of 20 μg/well for soluble tau fractions and 4 μg/well for insoluble tau fractions. Wells were rinsed twice, and then R1 was diluted (0.1 μg/ml) in blocking reagent and added to each well for 1.5 hours. Wells were rinsed 3 times and incubated for 1.5 hrs with goat anti-rabbit antibody conjugated to horseradish peroxidase (Vector Labs, PI-1000) diluted (0.2 μg/ml) in blocking reagent. The wells were rinsed 3 times, signal detected by developing with 3,3′,5,5′-tetramethylbenzidine (TMB) for 10–15 min and then the reaction was stopped using 3.5% sulfuric acid. The absorbance of TMB signal was measured at 450 nm.

### Dot Blot

Brain extracts were analyzed as previously described^23^ with the following exceptions. Samples were spotted onto the nitrocellulose membrane using a Whatman minifold I dot blot apparatus. The membranes were blocked, probed with TOC1 (oligomeric tau, diluted 1:5,000, Dr. Kanaan laboratory), and R1 (a pan-tau rabbit polyclonal antibody, diluted 1:20,000) and the appropriate Licor secondary antibodies (diluted 1:20,000), and imaged using the Licor Odyssey system. The signal intensity measurements for each dot were expressed as the ratio of oligomeric tau (TOC1 signal) per total tau (R1 signal). The Licor Odyssey system provides the advantages of dual-color quantitative blotting with a larger dynamic range and higher sensitivity than X-ray film and chemiluminescence. In a separate set of dot blots, the membranes were probed with either T22 antibody (1:1000, Millipore), and an anti-rabbit HRP conjugated secondary antibody diluted (1:20000) and detected using chemiluminescence and film.

### Histopathology and Histochemistry

Immunohistochemistry was performed using the Ventana BenchMark Ultra automated platform (see Supplementary Methods for a detailed description).

### Aβ Preparation

Aβ_42_ was prepared from synthetic peptide from the Teplow lab, as previously described^58^,^64^ (see Supplementary Methods for a detailed description).

### Assessment of Tau Entrance into Neurons

Primary cultures of hippocampal neurons were obtained from C57/BL6 mice, and eGFP-expressing mice, as previously described^65^,^66^. Before conjugation, monomeric tau was incubated with 1 mM dithiothreitol (DTT/PBS) for 10 min at 60 °C. Both oligomeric and monomeric preparations were purified with *Amicon* Ultra Centrifugal Filter (10 KDa). Tau preparations were then labeled with the IRIS 5-NHS active ester dye (IRIS 5; λ_ex_: 633 nm; λ_em_: 650–700 nm; Cyanine Technology, Turin, Italy) according to manufacturer’s protocol. Briefly, tau solutions (2 μM in PBS) were mixed with 6 mM IRIS 5 in dimethyl sulfoxide for 4 hrs in the dark under mild shaking conditions. After this time, labeled tau was purified with Vivacon 500 ultrafiltration spin columns (Sartorius Stedim Biotech GmbH, Goettingen, Germany) and then resuspended in PBS and used at final concentration of 100 nM. For time lapse confocal imaging experiment, hippocampal neurons from eGFP mice were cultured for 14 days *in vitro* (DIV) before being exposed to IRIS-5-labelled tau preparations for 40 min in Tyrode’s solution at 37 °C. Immunocytochemistry was also performed in 14 DIV neurons treated with IRIS 5-labeled monomeric or oligomeric tau for 3, 6 and 18 hrs. After two washes in PBS, cells were fixed with paraformaldehyde (4% in PBS; Sigma) for 15 min at RT. After being permeabilized (15-min of incubation with 0.3% Triton X-100 [Sigma] in PBS), cells were incubated for 20 min with 0.3% bovine serum albumin in PBS to block nonspecific binding sites and then overnight at 4 °C with the antibody anti-microtubule associated protein-2 (MAP-2, Immunological Sciences, Rome, Italy). The next day, cells were incubated for 90 min at room temperature with the secondary antibodies Alexa Fluor 488 donkey anti-mouse (1:1,000; Invitrogen). Images (1024 × 1024 pixels) were acquired at 63× magnification with a confocal laser scanning system (TCS-SP2; Leica Microsystems) and an oil-immersion objective (numerical aperture 1.4; physical pixel size 233 nm). For time lapse imaging, confocal Z-stacks were acquired every 5 min in order to study tau internalization. All experiments were repeated at least 3 times. The operator was blind to the study conditions.

### Statistical Analyses

Experiments were performed blinded with regards to vehicle or preparations. Results were expressed as the mean ± the standard error of the mean (SEM) (level of significance at p < 0.05). Results were analyzed by two-tailed Student’s t test, or ANOVA plus post-hoc multiple comparisons test using Prism (GraphPad) software with treatment condition as main effect. Behavioral experiments were designed in a balanced fashion. For each condition, mice were trained and tested in three to four separate sets of experiments.

## Additional Information

**How to cite this article**: Fá, M. *et al.* Extracellular Tau Oligomers Produce An Immediate Impairment of LTP and Memory. *Sci. Rep.* **6**, 19393; doi: 10.1038/srep19393 (2016).

## Supplementary Material

Supplementary Information
