# Supplementary material for: Extracellular Tau Oligomers Produce An Immediate Impairment of LTP and Memory
Source: Sci Rep. 2016 Jan 20;6:19393. doi: 10.1038/srep19393 (PMC4726138; doi:10.1038/srep19393)
Supplement: Supplementary Information [file srep19393-s1.doc]

## SUPPLEMENTARY INFORMATION

**TITLE: EXTRACELLULAR TAU OLIGOMERS PRODUCE AN IMMEDIATE IMPAIRMENT OF LTP AND MEMORY**

M. Fá^1,*^, D. Puzzo^1,2,*^, R. Piacentini^3^, A. Staniszewski^1^, H. Zhang^1^, M.A. Baltrons^1,4^, D.D. Li Puma^3^, I. Chatterjee^1,5^, J. Li^1,6^, F. Saeed^1^, H.L. Berman^1^, C. Ripoli^3^, W. Gulisano^2^, J. Gonzalez^7^, H. Tian^8^, J.A. Costa^1^, P. Lopez^5^, E. Davidowitz^5^, W.H. Yu^1^, V. Haroutunian^9^, L.M. Brown^10^, A. Palmeri^2^, E.M. Sigurdsson^11^, K.E. Duff^1^, A.F. Teich^1^, L.S. Honig^1^, M. Sierks^7^, J.G. Moe^4^, L. D’Adamio^12^, C. Grassi^3^, N.M. Kanaan^13^, P.E. Fraser^14^, O. Arancio^1,#^


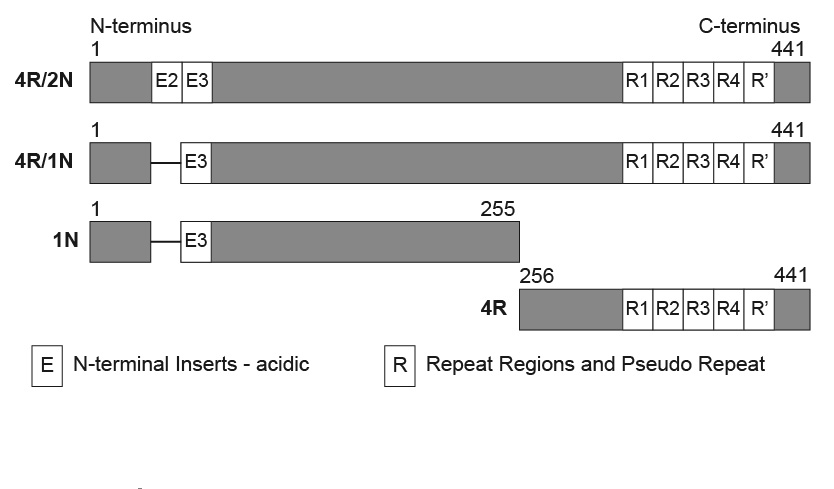


**Supplementary Figure S1. Schematic of tau constructs used and their nomenclature.** Six tau isoforms are encoded by the MAPT gene in the brain due to alternative splicing of exons 2 and 3 that encode N-terminal inserts (N) and exon 10 encoding the second microtubule binding repeat (R). The nomenclature is based on the domains included in the final protein that can have either zero, one or two N units, and three or four R units. The two longest tau isoforms, 4R/2N and 4R/1N, differ by the size of the inserts at the N-terminal (1N, 29 amino acids; 2N, 58 amino acids) and possess 4 repeats (4R) in the microtubule binding domain at their C-terminal side [1](#_ENREF_1). The numbering scheme is based on the longest isoform, 4R/2N, containing 441 amino acids.

**Supplementary Figure S2. Bilateral Injections of Recombinant Tau Oligomers into the Dorsal Hippocampi did not Affect Cued Fear Conditioning, Sensory Threshold, or Performance with the Visible Platform Task and Open Field Test. (A)** Freezing responses before (Pre) and after (Post) the auditory cue were the same among vehicle- (n = 18), and 22.95 µg/ml oTau 4R/2N- (n = 11) infused mice in the cued conditioning test. p > 0.05. **(B)** No difference was detected during assessment of the sensory threshold in vehicle (n = 18) and 22.95 µg/ml oTau 4R/2N-infused mice (n = 11). p > 0.05. **(C-D)** Testing with the visible platform task for assessment of visual-motor-motivational deficits did not reveal any difference for both speed (C) and time to the platform (D) between vehicle- (n = 11) and 22.95 µg/ml oTau 4R/2N-infused mice (n = 13). p > 0.05. **(E-F)** Open field testing in vehicle- and 22.95 µg/ml oTau 4R/2N-infused mice showed a similar percentage of time spent in the center compartment (E) and the number of entries into the center compartment (F) indicating that they had no differences in exploratory behavior (vehicle: n = 11, oTau 4R/2N: n = 13). p > 0.05. All data shown are mean ± SEM.


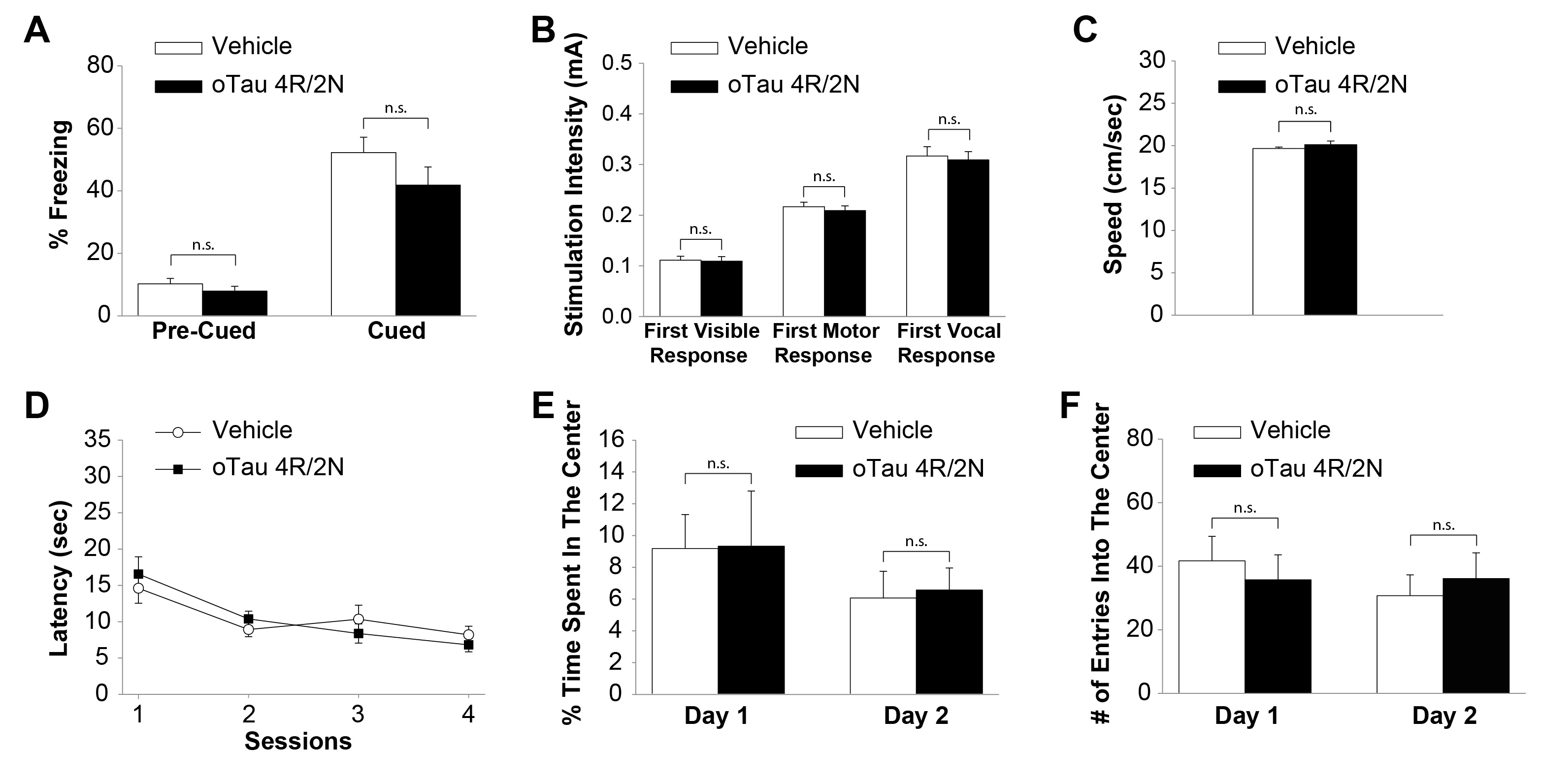


**A**

**
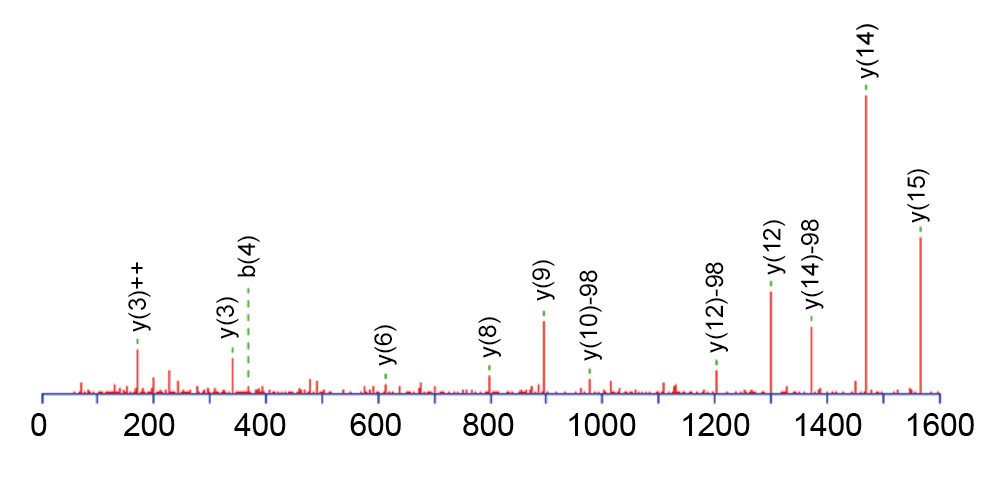
**

**B**

**
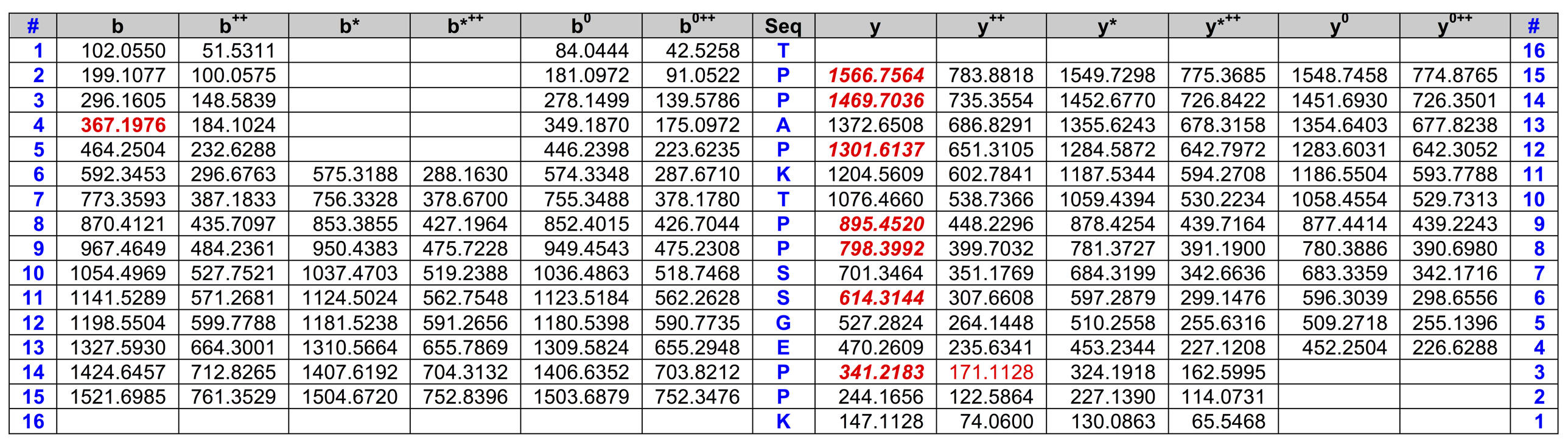
**

**C**

**
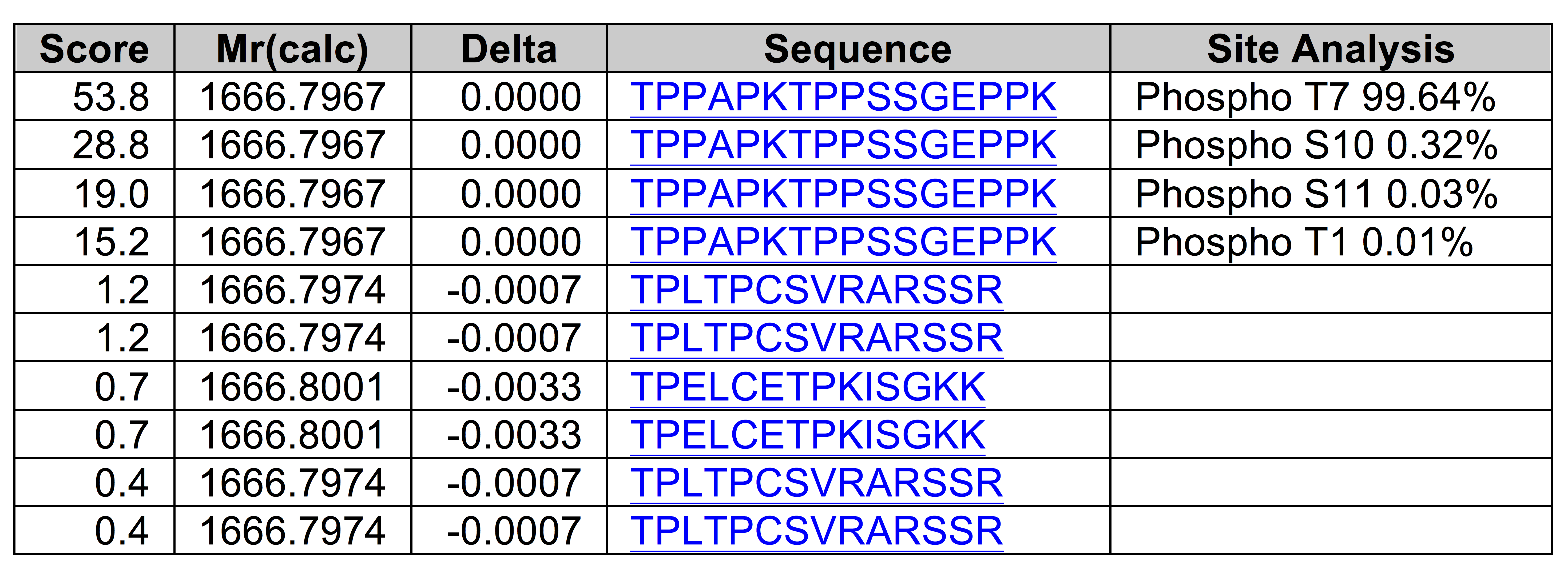
**

**Supplementary Figure S3. Proteomic Assessment of Phosphorylation Sites in the AD-Tau Preparation. (A-C)** Example MS/MS data for the peptide 175_TPPAPKpTPPSSGEPPK_190 from tau (identified in the NCBI non-redundant database as protein accession (gi) numbers 294862261, 6754638, 8400711, 8400715, 178557736, 294862258, 322303720, 32230374). Spectrum was acquired in data-dependent acquisition (DDA) mode on a Synapt G2 HDMS as analyzed by Mascot software. This peptide had a Mascot ion score of 54, precursor mass of 1666.79, charge state of +3, precursor mass error of 0.06 ppm, and product RMS error of 9 ppm. For this and the following panels, symbols (*) indicate loss of NH_3_, (0) indicates the loss of H_2_O, and ions were recorded as singly charged except if doubly charged (++). (A) Product ion spectrum with y-series and b-series ions labeled. Neutral losses of 98 indicated on the spectrum represent loss of H_3_PO_4_ from phosphorylated peptide fragment ions. (B) Table of detected masses as annotated by Mascot. Bold italic red indicates that the ion series contributed to the peptide score. Bold red indicates the number of matches in the ion series is greater than would be expected by chance alone, suggesting that the ion series is present in the spectrum. Non-bold red means that the number of matches in the ion series is no greater than would be expected by chance. Masses indicated in black type were not detected in the spectrum. (C) Alternate possible predicted sequence and Mascot site analysis indicating phosphorylation is probably on residue T7. **(D-F)** Example MS/MS data for the peptide 396_SPVVSGDTpSPR_406 from Microtubule-associated protein tau (sequence present in isoforms 1-8 represented by NCBInr gi numbers 294862261, 6754638, 8400711, 8400715, 178557736, 294862258, 322303720, 322303747). Spectrum was acquired in data-dependent acquisition (DDA) mode on a Synapt G2 HDMS as analyzed by Mascot software. This peptide had a Mascot ion score of 43, precursor mass of 1180.51, charge state of +2, precursor mass error of 4.6 ppm, and product RMS error of 6 ppm. (D) Product ion spectrum with y-series and b-series ions labeled. Neutral losses of 98 indicated on the spectrum represent loss of H_3_PO_4_ from phosphorylated peptide fragment ions. (E) Table of detected masses as annotated by Mascot. Bold italic red indicates that the ion series contributed to the peptide score. Bold red indicates the number of matches in the ion series is greater than would be expected by chance alone, suggesting that the ion series is present in the spectrum. Non-bold red means that the number of matches in the ion series is no greater than would be expected by chance. Masses indicated in black type were not detected in the spectrum. (F) Mascot site analysis indicating phosphorylation is probably on residue S9 or T8.

**D**

**
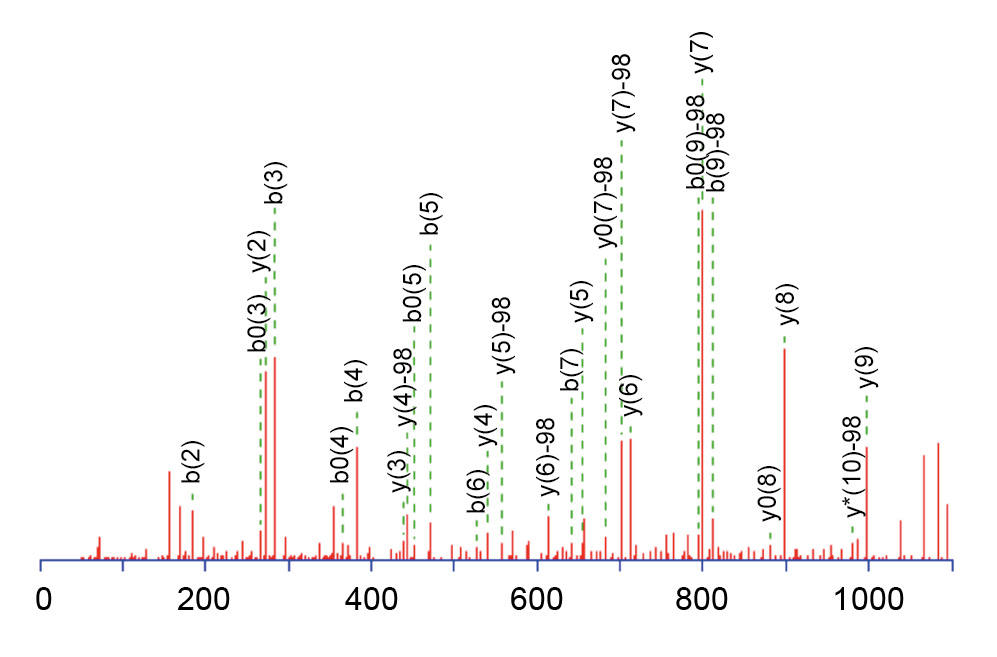
**

**E**

**
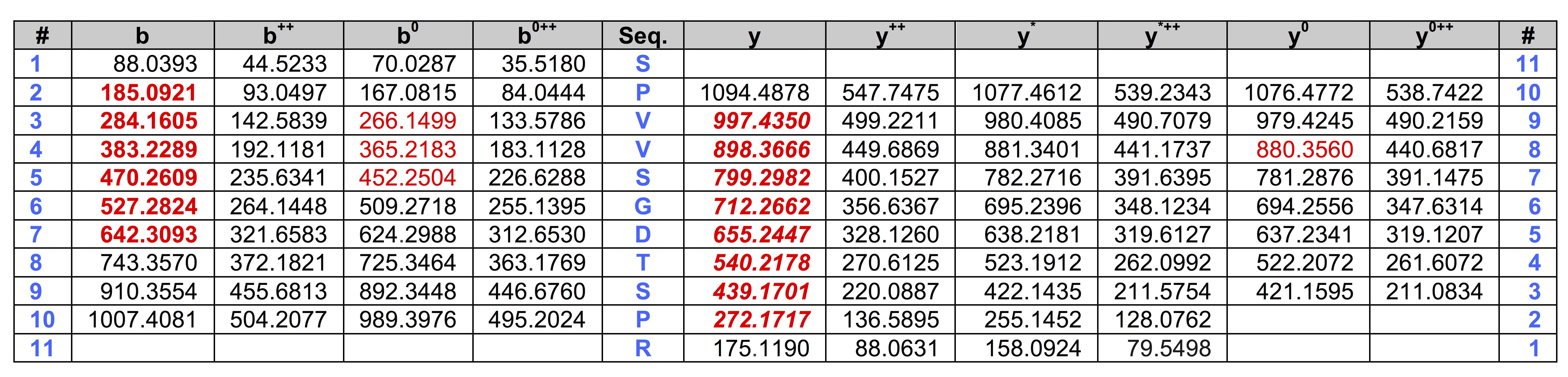
**

**F**

**
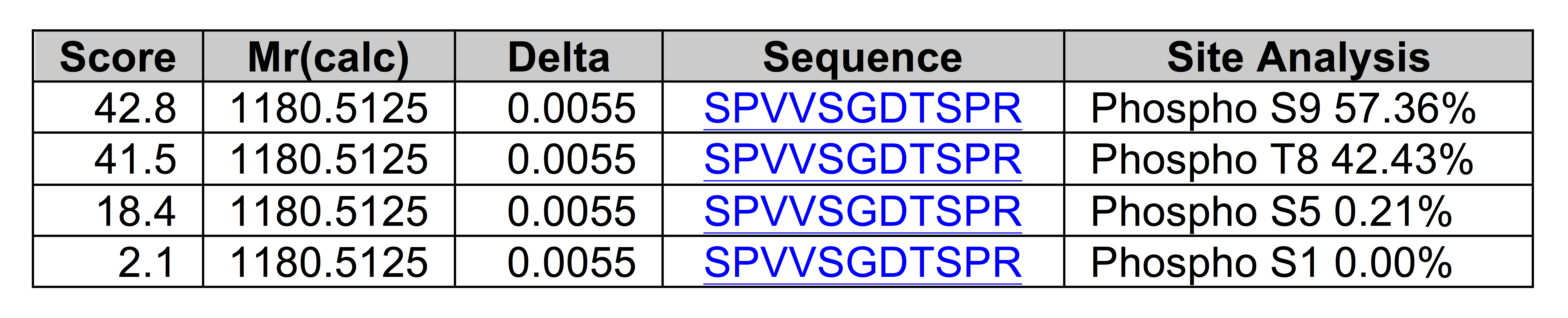
**


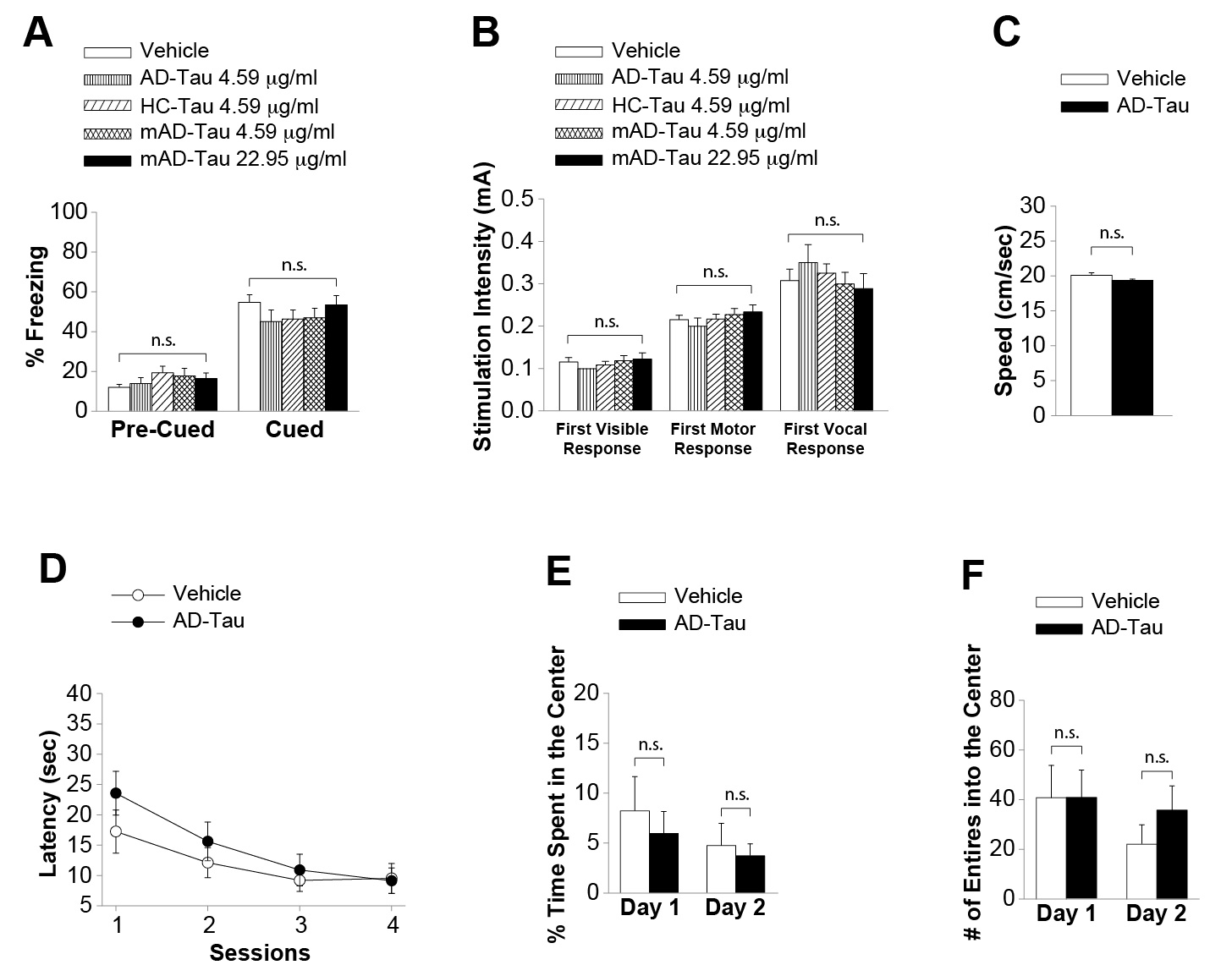


**Supplementary Figure S4. Cued Fear Memory, Sensory Threshold, Performance with the Visible Platform Task and Open Field Test, and Basal Synaptic Transmission are Normal following Bilateral Injections of a Preparation Enriched in Soluble Human Tau Derived from AD Patients. (A)** Freezing responses before (Pre) and after (Post) the auditory cue were the same among vehicle- (n = 13), AD-Tau- (4.59 µg/ml, n = 9), HC-Tau- (4.59 µg/ml; n = 12) or mAD-Tau- (both at 4.59 µg/ml and 22.95 µg/ml; n = 11 and 9 respectively) infused mice in the cued conditioning test. p > 0.05. Specimens from patients 34, 36 and 49 for AD and 28, 33 and 41 for HC (Table S1). **(B)** No difference was detected between groups during assessment of the sensory threshold in vehicle- and AD-Tau infused mice. Vehicle: n = 13, AD-Tau (4.59 µg/ml) n = 9, HC-Tau (4.59 µg/ml) n = 12, mAD-Tau (4.59 µg/ml and 22.95 µg/ml) n = 11 and 9, respectively, p > 0.05. Specimens from patients 34, 36 and 49 for AD and 28, 33 and 41 for HC (Table S1). **(C-D)** Testing with the visible platform task for assessment of visual-motor-motivational deficits did not reveal any difference in speed (C) and time to the platform (D) between vehicle- and AD-Tau infused mice (n = 8 per each group, respectively). p > 0.05. Specimens from patients 35, 37 (Table S1). **(E-F)** Open field testing in vehicle- and AD-Tau infused mice showed a similar percentage of time spent in the center compartment (E) and the number of entries into the center compartment (F) (n = 8 per each group; p > 0.05 for both), indicating that they had no differences in exploratory behavior. Specimens from patients 35, 37 (Table S1).

**Supplementary Figure S5. Cued Fear Memory, Sensory Threshold, Performance with the Visible Platform Task and Open Field Test, and Basal Synaptic Transmission are Normal in Mice Expressing Non-Mutated Human Tau Gene. (A)** Immunohistochemistry for hyper-phosphorylated human tau shows no staining in brain sections from adult (10-11 months of age) control mice (I). However, prominent staining was visible in adult (II) and aged (18 months) (III) hTau mice. A Bielschowsky silver stain (which highlights NFTs in AD patients) shows a normal staining pattern in adult control (IV) and hTau (V) mice. In contrast, aged hTau mice (VI) show occasional NFTs (arrow head - NFT is shown larger in magnified sub-panel). Interestingly, aged hTau mice also have neuronal processes that stain more prominently with the Bielschowsky stain. **(B)** BST at the CA3-CA1 connection of slices from 10- to 11-month-old hTau mice was similar to control littermates (n = 9 slices from 6 mice per group for hTau; and 7 slices from 6 mice for controls; ANOVA: p > 0.05). **(C)** Freezing responses before (Pre) and after (Post) the auditory cue were the same in controls (n = 9) and hTau (n = 13) mice in the cued conditioning test. p > 0.05. **(D)** No difference was detected between controls and hTau mice during assessment of the sensory threshold (n = 9 for controls and 13 for hTau mice). p > 0.05. **(E-F)** Testing with visible platform task for assessment of visual-motor-motivational deficits did not reveal any difference in speed (E) and time to the platform (F) between controls and hTau mice (n = 9 for controls and 13 for hTau mice). p > 0.05. **(G-H)** Open field testing in controls and hTau mice showed a similar percentage of time spent in the center compartment (G) and the number of entries into the center compartment (H) (p > 0.05 for both), indicating that they had no differences in exploratory behavior (n = 8 for controls and 9 for hTau mice). **(I)** Representative examples of non-reducing SDS-PAGE analysis of hTau-p and C-p. Numbers at the bottom of the WB correspond to fraction samples obtained during chromatography. All data shown are mean ± SEM.


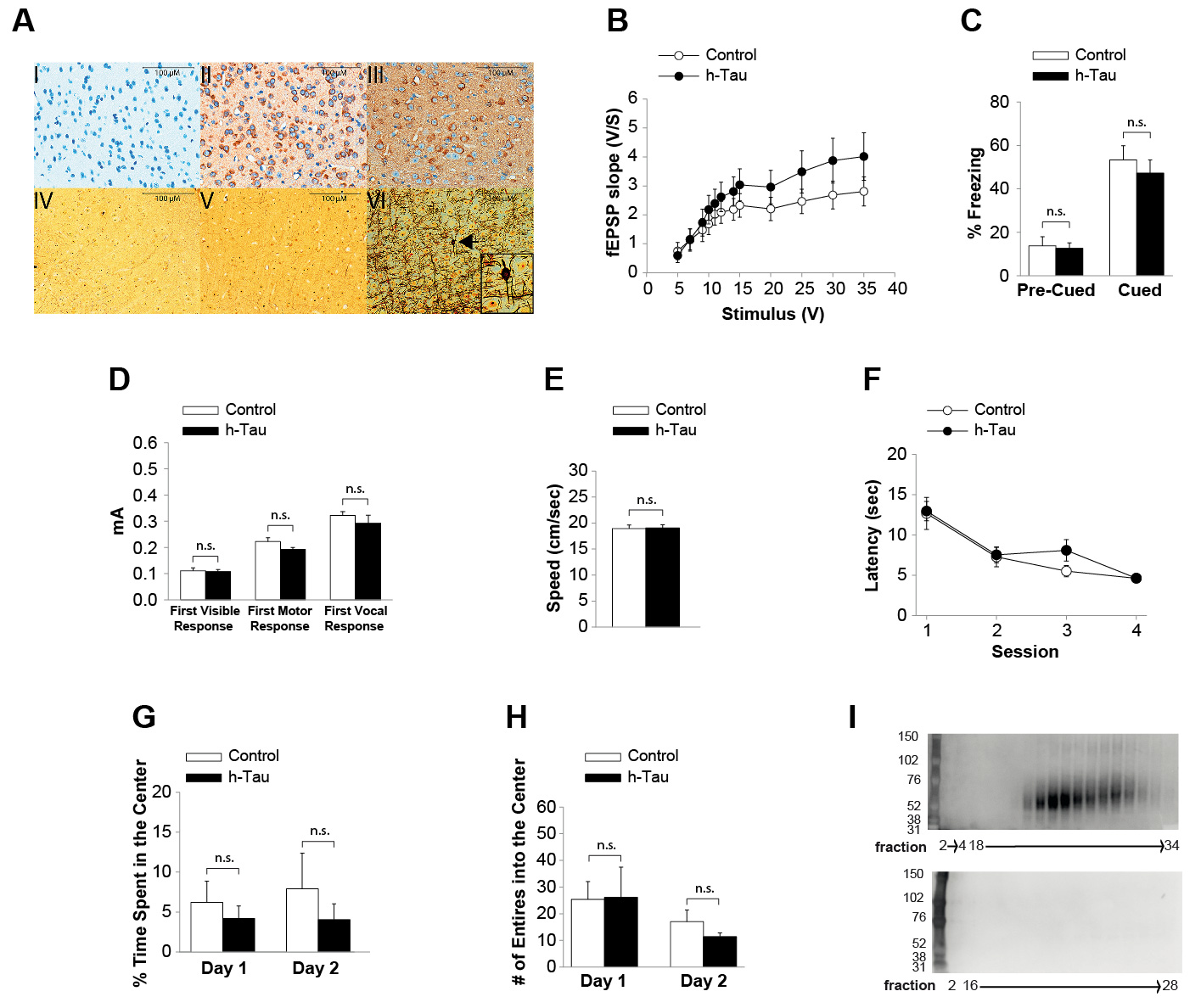


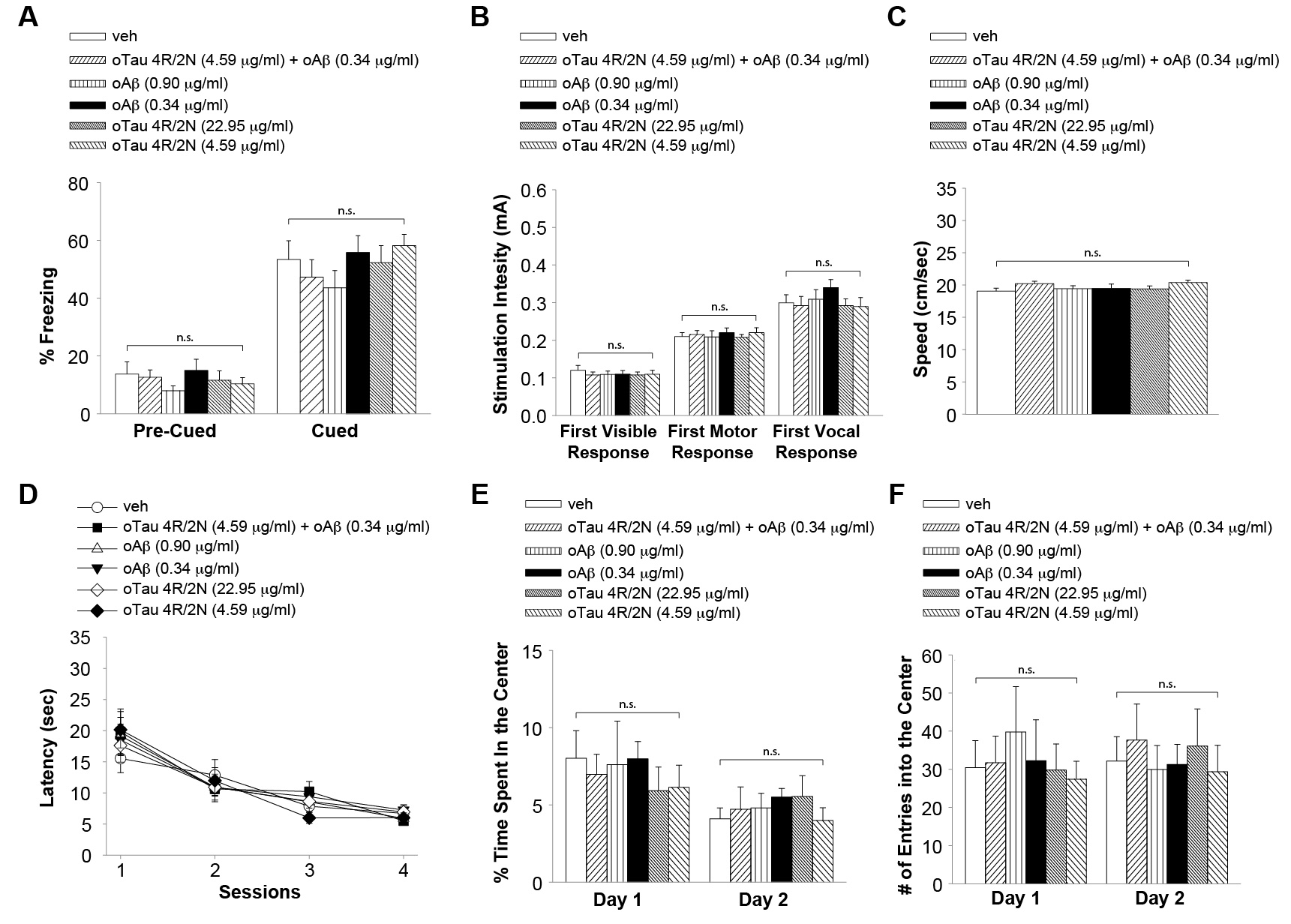


**Supplementary Figure S6.** **Bilateral Injections of oTau Concurrently with oAβ into the Dorsal Hippocampi did not Affect Cued Fear Conditioning, Sensory Threshold, or Performance with the Visible Platform Task and Open field Test. (A)** Freezing responses before (Pre) and after (Post) the auditory cue were the same among vehicle- (n = 10), 4.59 µg/ml oTau 4R/2N + 0.34 µg/ml oAβ (n = 13), 0.90 µg/ml oAβ (n = 11), 0.34 µg/ml oAβ (n=11), 22.95 µg/ml oTau 4R/2N (n = 13), and 4.59 µg/ml oTau 4R/2N (n = 10) infused mice in the cued conditioning test. ANOVA: p > 0.05. **(B)** No difference was detected between groups during assessment of the sensory threshold in vehicle- (n = 10), 4.59 µg/ml oTau 4R/2N + 0.34 µg/ml oAβ- (n = 13), 0.90 µg/ml oAβ- (n = 11), 0.34 µg/ml oAβ- (n=11), 22.95 µg/ml oTau 4R/2N- (n = 13), and 4.59 µg/ml oTau 4R/2N- (n = 10) infused mice. ANOVA: p > 0.05. **(C-D)** Testing with the visible platform task for assessment of visual-motor-motivational deficits did not reveal any difference in speed (C) and time to the platform (D) between vehicle- (n=11), 4.59 µg/ml oTau 4R/2N + 0.34 µg/ml oAβ- (n=11), 0.90 µg/ml oAβ- (n=9), 0.34 µg/ml oAβ- (n=13), 22.95 µg/ml oTau 4R/2N- (n = 9) and 4.59 µg/ml oTau 4R/2N- (n = 11) infused mice. ANOVA: p > 0.05. **(E-F)** Open field testing showed a similar percentage of time spent in the center compartment (E) and the number of entries into the center compartment (F) in vehicle- (n = 10), 4.59 µg/ml oTau 4R/2N + 0.34 µg/ml oAβ- (n = 9), 0.90 µg/ml oAβ- (n = 9), 0.34 µg/ml oAβ- (n = 7), 22.95 µg/ml oTau 4R/2N- (n = 9), and 4.59 µg/ml oTau 4R/2N- (n = 9) infused mice in the cued conditioning test. ANOVA: p > 0.05, indicating that they had no differences in exploratory behavior. All data shown are mean ± SEM.

**Supplementary Tables**

| **subject #** | **diagnosis** | **age** | **gender** | **PMI** | **Braak #** | **CERAD #** | **NIA-R #** |
| --- | --- | --- | --- | --- | --- | --- | --- |
| 1 | HC | 36 | M | 9:00 | 0 | 0 | 0 |
| 2 | AD | 44 | M | 20:08 | VI | C | HIGH |
| 3 | HC | 54 | F | 15:40 | 0 | 0 | 0 |
| 4 | HC | 57 | F | 9:40 | 0 | 0 | 0 |
| 5 | HC | 57 | M | 6:50 | 0 | 0 | 0 |
| 6 | AD | 61 | M | 5:00 | VI | C | HIGH |
| 7 | AD | 62 | F | 23:40 | VI | undetermined | HIGH |
| 8 | HC | 64 | F | 11:30 | 0 | 0 | 0 |
| 9 | AD | 65 | M | 9:50 | VI | undetermined | HIGH |
| 10 | AD | 67 | M | 25:20 | VI | C | HIGH |
| 11 | AD | 68 | M | 7:40 | VI | C | HIGH |
| 12 | HC | 70 | M | 23:50 | 0 | 0 | undetermined |
| 13 | HC | 71 | M | 21:37 | 0 | 0 | undetermined |
| 14 | HC | 72 | M | 4:30 | 0 | 0 | 0 |
| 15 | AD | 72 | M | 9:00 | VI | C | HIGH |
| 16 | HC | 73 | F | 3:00 | 0 | 0 | undetermined |
| 17 | HC | 74 | M | 11:50 | 0 | 0 | undetermined |
| 18 | AD | 74 | F | 23:09 | VI | C | HIGH |
| 19 | HC | 78 | M | 8:00 | 0 | 0 | 0 |
| 20 | HC | 78 | M | 8:00 | 0 | 0 | 0 |
| 21 | AD | 78 | F | 41:30 | VI | undetermined | HIGH |
| 22 | AD | 79 | F | 11:40 | VI | undetermined | HIGH |
| 23 | HC | 83 | M | 16:50 | 0 | 0 | undetermined |
| 24 | AD | 83 | F | 5:00 | VI | B | HIGH |
| 25 | HC | 89 | M | 9:30 | III | A | LOW |

**Supplementary Table S1** **Brain Bank Sample Characteristics.** A list of frozen post-mortem tissue samples from healthy control individuals (HCs) or neuropathologically confirmed AD cases. All individuals were characterized for their Braak and Braak stage (Braak # 0-VI), Consortium to Establish a Registry for Alzheimer’s Disease (CERAD) Neuropsychological Battery score (C for AD patients, not evaluable for HCs), and National Institute of Aging – Reagan Institute rating (NIA-R) (high for the AD patients, 0 for HCs). AD patients had no other diseases that might have contributed to the clinical deficits. De-identified specimens include AD and HCs. HC: range 36-89 yrs, average: 68.28 ± 3.65; probable AD: range: 44-83 yrs, average: 68.45 ± 3.25 yrs. PMI: post-mortem interval.

| Phosphorylation site | Phosphopeptide | m/z | Measured Mass (Da) | Mascot site analysis score (%) | Mascot peptide ion score | Reference |
| --- | --- | --- | --- | --- | --- | --- |
| 181 | 175_TPPAPKpTPPSSGEPPK_190 | 556.61^3+^ | 1666.80 | 99.6 | 54 | This study |
| 181 | 175_TPPAPKpTPPSSGEPPK_190 | 556.61^3+^ | 1666.80 | - | - | [^2^](#_ENREF_2) |
|  |  |  |  |  |  |  |
| 404 | 396_SPVVSGDTpSPR_406 | 591.27^2+^ | 1180.52 | 57.4 | 43 | This study |
| 404 | 396_SPVVSGDTpSPR_406 | 591.26^2+^ | 1180.51 | - | - | [^2^](#_ENREF_2) |

**Supplementary Table S2** **Some Phosphorylation Sites Detected, their Putative Locations and a Comparison to Data Previously Reported in the Literature.** Tau was identified in the NCBI non-redundant database as protein accession (gi) numbers 294862261, 6754638, 8400711, 8400715, 178557736, 294862258, 322303720, 32230374. Our purification technique preserves the phospho-epitopes of adult human tau suggesting that our extraction method produces samples amenable to analysis by mass spectrometry and provides usable spectra of phosphorylated peptides, as shown on Figure S3A-F. The table compares our results with data in the literature and demonstrates that our results are essentially identical to those published by other groups. The sites identified, their assignment to particular residues, the m/z values and charge states were all identical to those reported by other groups [^2^](#_ENREF_2). These results demonstrate the effectiveness of a technique simpler than previous protocols [^3^](#_ENREF_3), yet still allowing efficient detection of phosphosites. The mass spectra collected compared favorably with spectra for these same peptides in public databases . These spectra demonstrated the potential for using this technique for studying phosphorylation in future more comprehensive studies. We also demonstrate that tau phosphorylation survived this perchloric acid treatment in a manner that retained compatibility with mass spectrometric identification. Finally, these findings confirm the stability of phosphosites to this extraction method, already shown with immunologic data [^6^](#_ENREF_6), through the use of mass spectrometry.

**SUPPLEMENTARY METHODS**

***Atomic Force Microscopy (AFM)***

For AFM sample preparation, a 10 μl aliquot of 10 μg/μl tau protein solution was deposited on a freshly cleaved mica substrate (Spruce Pine, NC), incubated for 5 min at room temperature, rinsed extensively with 0.2 μm filtered deionized water (18.1 MΩ, Millipore, MA), and dried under a gentle stream of N_2_ gas. All AFM height images were recorded in tapping mode with scan rates of 2.5 Hz and 512x512 pixels resolution with commercial AFMs in air at room temperature. AFM images for height distribution calculations were acquired using the Nanoscope IIIa AFM (Veeco, Santa Barbara, CA) equipped with oxide sharpened Si_3_N_4_ AFM tips (k = 40 N/m, f_o_ ~ 300kHz) (Model: OTESPA, Veeco, Santa Barbara, CA) and analyzed with the Scanning Probe Imaging Processor software (SPIP, Image Metrology) to generate height distribution plots, as previously described [^7^](#_ENREF_7). Presented topographic AFM images were recorded using the MFP-3D AFM (Asylum Research, Santa Barbara, CA) equipped with commercial microcantilevers (k = 2 N/m, f_o_ ~ 70kHz) (OMCL-AC240TS-W2, Olympus, Japan). Images are displayed 3-dimensionally without further processing using the implemented ARgyle Light 3D imaging module (Asylum Research, Santa Barbara, CA).

***Electrophysiological Studies***

Hippocampal slices (400 μm) were cut with a tissue chopper and maintained in an interface chamber at 29° C for 90 minutes prior to recording, as previously described [^8^](#_ENREF_8). Following assessment of basal synaptic transmission by plotting the stimulus voltages against slopes of field Excitatory Post-Synaptic Potentials (fEPSP); baseline was recorded every minute at an intensity that evoked a response 35% of the maximum evoked response, baseline was recorded every minute at an intensity that evoked a response 35% of the maximum evoked response. Slices were perfused for 20 min with different tau and Aβ preparations or vehicle, and LTP was induced using a theta-burst stimulation (4 pulses at 100 Hz, with the bursts repeated at 5 Hz and each tetanus including 3 ten-burst trains separated by 15 sec). Responses were measured as fEPSP slopes expressed as percentage of baseline.

***Behavioral Studies***

*Intracerebral tau and Aβ infusion.* After 5-7 days from the implant of cannulas onto dorsal hippocampi [^8^](#_ENREF_8), mice were bilaterally infused with tau or Aβ preparations or vehicle in a final volume of 1 μl over 1 minute with a microsyringe connected to the cannulas via polyethylene tubing. Tau was infused at 180 and 20 minutes prior to the foot shock, whereas Aβ was infused at 20 minutes prior the foot shock. During infusion animals were handled gently to minimize stress. After infusion, the needle was left in place for another minute to allow diffusion. Mice were handled once a day for 3 days before behavioral assessment. After behavioral testing, a solution of 4% methylene blue was infused into the cannulas to check for the position of the cannulas, as described [^8^](#_ENREF_8).

*Fear conditioning (FC)* [^8^](#_ENREF_8). Mice were placed in a conditioning chamber for 2 minutes before the onset of a tone (Conditioning Stimulus, CS) (a 30 sec, 85 dB sound at 2800 Hz). In the last 2 sec of the CS, mice were given a 2 sec, 0.6 mA foot shock (Unconditioning Stimulus, US) through the bars of the grid-floor and left in the conditioning chamber for additional 30 sec. Freezing behavior (the absence of all movements except for those needed for breathing) was scored using FreezeView software. Contextual fear learning was evaluated 24 hrs after training by measuring freezing for 5 min in the chamber in which mice were trained. Cued fear learning, a type of memory depending upon amygdala function [^9^](#_ENREF_9), was assessed 24 hrs after contextual testing by placing mice in a novel context for 2 minutes (pre-CS test), after which they were exposed to the CS for 3 min (CS test). To determine whether the treatments affected sensory perception of the mice, threshold assessment was conducted as previously described [^8^](#_ENREF_8).

*2-day Radial Arm Water Maze (RAWM)* . During the first day of the protocol mice were trained to identify the platform location by alternating between a visible and a hidden platform in a goal arm, except that during the last 3 trials in which the platform was hidden. On the second day, in turn, all 15 trials were hidden. Entries to arms with no platform were counted as an error, and the animal was gently pulled back to the start arm. Failure to select an arm after 15 sec was also counted as an error and the mouse was returned to the start arm. The duration of each trial was up to 1 min. At the end of each trial mouse rested on the platform for 15 sec. The platform location was different for each mouse. Data were analyzed and displayed as averages of blocks of 3 trials per mouse. Controls for this task were performed with the visible platform and open field test [^8^](#_ENREF_8).

***Histopathology and Histochemistry***

Immunohistochemistry was performed using the Ventana BenchMark Ultra automated platform. Tissue sections were first deparaffinized using Ventana’s “ez-prep” solution. Antigen retrieval was performed by treatment at 95°C for 54 minutes using Ventana's CC1 (pH7.3) solution, followed by treatment with 0.3% hydrogen peroxide to block endogenous peroxidase. Tissue sections were then incubated in protein-free block (Biocare’s background sniper) for 15 min to inhibit the nonspecific binding of primary. Primary antibody (AT8 at 1:200, Thermo Scientific) was incubated for 32 min at room temperature. Detection was performed using Ventana’s ultraview DAB kit, and counterstaining with the Gill hematoxylin solution. For Bielschowsky staining, slides were first deparaffinized and hydrated with distilled water. They were then placed in 20% Silver Nitrate at 60° C for 15 min, rinsed, and then stained in ammoniacal silver solution for 30 min. Slides were then rinsed in tap water, washed in sodium thiosulfate solution for 2 min, rinsed in tap water, and then dehydrated and mounted with synthetic resin.

***Aβ Preparation***

Aβ_42_ was prepared from synthetic peptide from the Teplow lab, as previously described [^8^](#_ENREF_8). Briefly, lyophilized Aβ_42_ was resuspended in cold 1,1,1,3,3,3-hexafluoro-2-propanol (HFIP, Sigma) and aliquoted in polypropylene vials. After 24 hrs, the HFIP solution was allowed to evaporate in a fume hood until formation of a thin film of monomeric peptide at the bottom of the vials. Peptide films, dried under gentle vacuum, were stored in sealed vials at –20°C. Prior to use, following monomerization of Aβ through DMSO (Sigma), the peptide was sonicated for 10 minutes [^12^](#_ENREF_12). Aβ_42_ oligomers were obtained by incubating an aliquot of monomeric Aβ/DMSO solution in sterile phosphate buffer at 4°C overnight. Oligomerized Aβ peptide was diluted to the final concentration with vehicle immediately before the experiments.

**Supplementary References**

1 Himmler, A., Drechsel, D., Kirschner, M. W. & Martin, D. W., Jr. Tau consists of a set of proteins with repeated C-terminal microtubule-binding domains and variable N-terminal domains. *Molecular and cellular biology* **9**, 1381-1388 DOI: 10.1128/MCB.9.4.1381 (1989).

2 Hanger, D. P. *et al.* Novel phosphorylation sites in tau from Alzheimer brain support a role for casein kinase 1 in disease pathogenesis. *J. Biol. Chem.* **282**, 23645-23654, doi:10.1074/jbc.M703269200 (2007).

3 Hanger, D. P., Betts, J. C., Loviny, T. L. F., Blackstock, W. P. & Anderton, B. H. New phosphorylation sites identified in hyperphosphorylated tau (paired helical filament-tau) from Alzheimer's disease brain using nanoelectrospray mass spectrometry. *J. Neurochem.* **71**, 2465-2476 DOI:10.1046/j.1471-4159.1998.71062465.x (1998).

4 Bodenmiller, B. *et al.* PhosphoPep-a database of protein phosphorylation sites in model organisms. *Nature Biotechnology* **26**, 1339-1340, doi:10.1038/nbt1208-1339 (2008).

5 Craig, R., Cortens, J. P. & Beavis, R. C. Open source system for analyzing, validating, and storing protein identification data. *Journal of Proteome Research* **3**, 1234-1242, doi:10.1021/pr049882h (2004).

6 Ivanovova, N., Handzusova, M., Hanes, J., Kontsekova, E. & Novak, M. High-yield purification of fetal tau preserving its structure and phosphorylation pattern. *J Immunol Methods* **339**, 17-22, doi:10.1016/j.jim.2008.07.014 (2008).

7 Wang, M. S., Zameer, A., Emadi, S. & Sierks, M. R. Characterizing antibody specificity to different protein morphologies by AFM. *Langmuir* **25**, 912-918, doi: 10.1021/la802591410.1021 (2009).

8 Puzzo, D. *et al.* Picomolar amyloid-beta positively modulates synaptic plasticity and memory in hippocampus. *The Journal of neuroscience : the official journal of the Society for Neuroscience* **28**, 14537-14545, doi:10.1523/JNEUROSCI.2692-08.2008 (2008).

9 Phillips, R. G. & LeDoux, J. E. Differential contribution of amygdala and hippocampus to cued and contextual fear conditioning. *Behavioral neuroscience* **106**, 274-285 doi:10.1037/0735-7044.106.2.274 (1992).

10 Alamed, J., Wilcock, D. M., Diamond, D. M., Gordon, M. N. & Morgan, D. Two-day radial-arm water maze learning and memory task; robust resolution of amyloid-related memory deficits in transgenic mice. *Nature protocols* **1**, 1671-1679, doi:10.1038/nprot.2006.275 (2006).

11 Fiorito, J. *et al.* Synthesis of quinoline derivatives: Discovery of a potent and selective phosphodiesterase 5 inhibitor for the treatment of Alzheimer's disease. *Eur J Med Chem* **60C**, 285-294, doi:10.1016/j.ejmech.2012.12.009 (2012).

12 Fa, M. *et al.* Preparation of oligomeric beta-amyloid 1-42 and induction of synaptic plasticity impairment on hippocampal slices. *Journal of visualized experiments : JoVE*, **41** doi:10.3791/1884 (2010).
